# The *white* gene as a transgenesis marker for the cricket *Gryllus bimaculatus*

**DOI:** 10.1093/g3journal/jkae235

**Published:** 2024-10-15

**Authors:** Emmanuel Gonzalez-Sqalli, Matthieu Caron, Benjamin Loppin

**Affiliations:** Laboratoire de Biologie et Modélisation de la Cellule, École Normale Supérieure de Lyon, CNRS UMR5239, Université Claude Bernard Lyon 1, 9 rue du Vercors, 69007 Lyon, France; Laboratoire de Biologie et Modélisation de la Cellule, École Normale Supérieure de Lyon, CNRS UMR5239, Université Claude Bernard Lyon 1, 9 rue du Vercors, 69007 Lyon, France; Laboratoire de Biologie et Modélisation de la Cellule, École Normale Supérieure de Lyon, CNRS UMR5239, Université Claude Bernard Lyon 1, 9 rue du Vercors, 69007 Lyon, France

**Keywords:** *Gryllus bimaculatus*, cricket, insect transgenesis, *white*, *piggyBac*, *Minos*, *PhiC31* integrase, eye, 3xP3, CenH3, centromere

## Abstract

The cricket *Gryllus bimaculatus* is an emerging model insect of the order Orthoptera that is used in a wide variety of biological research themes. This hemimetabolous species appears highly complementary to *Drosophila* and other well-established holometabolous models. To improve transgenesis applications in *G. bimaculatus*, we have designed a transformation marker gene inspired from the widespread *Drosophila* mini-*white*^+^**. Using CRISPR/Cas9, we first generated a loss-of-function mutant allele of the *Gb-white* gene (*Gb-w*), which exhibits a white eye coloration at all developmental stages. We then demonstrate that transgenic insertions of a *piggyBac* vector containing a *3xP3-Gb-w^+^* cassette rescue eye pigmentation. As an application, we used this vector to generate *G. bimaculatus* lines expressing a centromeric histone H3 variant (CenH3.1) fused to EGFP and validated EGFP-CenH3.1 detection at cricket centromeres. Finally, we demonstrate that *Minos*-based germline transformation and site-specific plasmid insertion with the *ΦC31* integrase system function in *G. bimaculatus*.

## Introduction

The two-spotted cricket *Gryllus bimaculatus* is an orthopteran insect (suborder Ensifera, family Gryllidae) that has recently emerged as a representative model of hemimetabolous species (Horch *et al.* 2017). Hemimetabolous insects, which do not have a larval stage, are still largely under-represented in the field of functional genetics in comparison with holometabolous model species such as *Drosophila melanogaster* (Diptera), *Tribolium castaneum* (Coleoptera), *Bombyx mori* (Lepidoptera), or *Nasonia vitripennis* (Hymenoptera), for instance. *G. bimaculatus* is a highly prolific and easily reared cricket that can be maintained at constant temperature all year round without diapause. The recent sequencing and annotation of the 1.66 Gb *G. bimaculatus* genome sequence (Ylla *et al.* 2021) and the amenability of large cricket eggs for microinjection (Barry *et al.* 2019) ideally position this species for the improvement of currently available genetic tools. These include for instance embryo RNAi (Miyawaki *et al.* 2004), parental RNAi (Mito *et al.* 2005), Zinc-Finger nucleases and TALENs-targeted mutagenesis (Watanabe *et al.* 2012), CRISPR/Cas9-targeted mutagenesis (Nakamura *et al.* 2022; Bai *et al.* 2023; Inoue *et al.* 2023), and CRISPR/Cas9-mediated nonhomologous transgene insertion (Matsuoka *et al.* 2021). The first *piggyBac*-mediated germline transformation in *G. bimaculatus* was reported in 2010 (Nakamura *et al.* 2010). These transgenic lines were isolated based on the expression of their cargo gene which produced a fluorescent protein in embryos. More recently, a *piggyBac* vector with the *3xP3-mCherry* eye marker was successfully used in crickets, but not documented (Watanabe *et al.* 2018). The design of a nonfluorescent, easily detected marker, as well as the improvement of currently available germline transformation techniques could encourage the use of this highly promising model in functional genetics.

The historical success of *D. melanogaster* as a model insect lies in a powerful combination of its intrinsic biological features and a unique arsenal of genetic tools. These include invaluable balancer chromosomes, a large collection of mapped chromosomal aberrations and a rich diversity of visible markers, to mention a few. The establishment of efficient *P*-mediated transgenesis in the 80s represented a major breakthrough for the model and more generally for animal genetics (Rubin and Spradling 1982; 1983). Although the eye pigmentation gene *rosy* was originally used as a visible marker to detect transgenic flies (Rubin and Spradling 1982), the highly sensitive and easily detectable *white* marker became soon after rapidly popular and is still the most widely used transgenesis marker in *D. melanogaster*. The *Drosophila white (w)*, *scarlet (st)*, and *brown (bw)* genes encode transmembrane guanine transporters of the ATP-binding cassette (ABC) family (Ewart and Howells 1998). In the *D. melanogaster* eye, W and St proteins form heterodimers that are required to import precursors of ommochrome pigment in pigment cell granules, which confer brown eye pigmentation (Tearle *et al.* 1989). The W protein also forms heterodimers with Bw to import the red pigment precursors pteridins (Dreesen *et al.* 1988; Ewart *et al.* 1994). Thus, W plays a central role in eye pigmentation and flies with a loss-of-function *w* allele have pure white eyes (Mackenzie *et al.* 1999).

The *w^+^* marker was first introduced in *P*-element based vectors as large genomic fragments (Gehring *et al.* 1984; Hazelrigg *et al.* 1984), but a shorter version of the *w^+^* marker, known as “mini-*white*”, where the large first intron is reduced in size, is now used in most classic *P*-based vectors (Klemenz *et al.* 1987; Thummel *et al.* 1988) or in site-specific transgene integration using the *ΦC31* integrase system (Bischof *et al.* 2007). When used in a *w* mutant background, transgenic *w^+^* expression restores eye pigmentation in a dose-dependent manner, ranging from pale orange to dark red color. The expression level of the *w^+^* marker depends on the type of promoter used in the vector, but also on the position of the insertion in the genome and the local chromatin context (Elgin and Reuter 2013). The sensitivity of the *w^+^* marker is useful to detect different segregating transgenic insertions in crosses, or to distinguish heterozygous from homozygous individuals for a given insertion.

Besides *D. melanogaster* and other *Drosophila* species, the *w^+^* marker for insect transgenesis has surprisingly only been used in two Tephritid fruit flies, the medfly *Ceratitis capitata* (Loukeris *et al.* 1995) and the oriental fruit fly *Bactrocera dorsalis* (Handler and McCombs 2000). Developing *w^+^* as a transgenesis marker in a new species indeed requires the identification and cloning of the *w* ortholog gene and the obtention of a *w* mutant allele. Moreover, in some insects, *w* appears essential for viability [*Helicoverpa armigera*, Lepidoptera (Khan *et al.* 2017); *Oncopeltus fasciatus*, Hemiptera (Reding and Pick 2020)], development [*Lygus hesperus*, Hemiptera (Brent and Hull 2019)], or reproduction [*Drosophila suzukii*, Diptera (Yan *et al.* 2020)], precluding its use for transgenesis. In addition, transgenesis in model and non-model species greatly benefited from the discovery of the *3xP3* artificial promoter (Berghammer *et al.* 1999; Horn *et al.* 2000). This small promoter, which contains 3 binding sites for the transcription factor Pax6/eyeless, allows the expression of fluorescent proteins (FP) in various larval organs as well as in adult eyes and ocelli of a wide range of insects. As fluorescence is strongly quenched by insect compound eye pigments, 3xP3-FP markers are preferably used in a *w* mutant genetic background (Berghammer *et al.* 1999). This could be particularly true for hemimetabolous insects that lack a larval stage, thus restricting 3xP3-FP detection to nymphal or adult eyes or ocelli.

Here, we report the use of the *G. bimaculatus white* gene as an alternative, efficient cricket transgenesis marker. Through CRISPR/Cas9 mutagenesis, we obtained and characterized a *G. bimaculatus white* (*Gb-w*) mutant strain and generated a versatile *piggyBac* transformation vector bearing a *Gb-w^+^* reporter cassette that rescues eye pigmentation. Finally, we show that *Minos*-based transgenesis can be used for germline transformation and we provide evidence that site-specific transgene insertion using the *ΦC31* integrase system functions in *G. bimaculatus*.

## Materials and methods

### Crickets

The wild-type *G. bimaculatus* strain used in this study was established from non-transgenic individuals of the *pGact-H2B-EGFP/+* strain (Nakamura *et al.* 2010). The “Hokudai *gwhite*” strain was previously described (Niwa *et al.* 1997; Watanabe *et al.* 2018). *pGact-H2B-EGFP/+* and “Hokudai *gwhite*” crickets were obtained from Dr. Cassandra Extavour (Harvard University). Crickets were reared in groups of 40–100 individuals in plastic cages with cardboard for hiding. Animals were kept at 29°C under a 12 h/12 h light–dark cycle in 400 L incubators (RUMED). First instar nymphs were fed with artificial fish food. Second instar nymphs to adults were fed ad libitum with powdered mouse food (Altromin) supplemented with cat food pellets. Water was supplied in *Drosophila* plastic vials or bottles capped with cellulose acetate flugs (Genesee Scientific #49-102). Transgenic lines were established by backcrossing injected crickets with *Gb-w^1^* individuals.

### Embryo microinjection

Needles for microinjection were made by pulling borosilicate glass capillaries (10 cm long, 1 mm external diameter, 0.5 mm internal diameter (World Precision Instruments, #BF100-50-10)) with a P-1000 micropipette puller (Sutter Instruments) using the following program: Heat = 470, Pull = 70, Velocity = 70, Delay = 110, Pressure = 500. After pulling, needles were beveled at a 25° angle using a BV-10 micropipette beveler (Sutter Instruments). Capillaries with an internal diameter of 10 ± 2 µm were used for injection, as described (Barry *et al.* 2019).

Eggs were collected by placing nests made from moistened paper napkins in cages containing about 50–100 adult crickets for up to 1 h. For microinjections, we used 100 mm Petri dish filled with 1.5% agarose in distilled water, Penicilline-Streptomycine 1X (Gibco #15070-063) colored with bromophenol blue. Eggs were aligned along parallel grooves made in the gel using a homemade glass structure. Once aligned, embryos were glued to the gel with 1% low melting point agarose (SIGMA #A9414-50G).

Embryos were microinjected using a FemtoJet 4i pump (Eppendorf) equipped with an InjectMan 4 micromanipulator system (Eppendorf). The system was installed on an AxioVert A1 inverted microscope (Zeiss) equipped with a ×10 objective. Injection mixes are detailed in Supplementary Table 1. After injection, embryos were placed in a humid chamber at 29°C until hatching.

### In vitro mRNA synthesis

For *piggyBac*-mediated transgenesis, hyPBase source was provided either as a helper plasmid [*DmHsp70-^i^hyPbase* (Eckermann *et al.* 2018) or *GbA3/4-^i^hyPBase* (this study)] or as in vitro synthesized mRNA. *hyPbase* mRNA was synthesized from a PCR amplicon of *GbA3/4-^i^hyPBase* plasmid DNA using the following primers OEG_073 GAAACTAATACGACTCACTATAGGGAGAGCCGCCACATGGGTAGTTCTTTAGACGATG and OEG_074 TATTTACAATTTTATGTCTTTATTTAATCTTT that contain the T7 promoter sequence. For *Minos*-mediated transgenesis, we used *Minos* transposase mRNA synthesized using the *pBlueSKMimRNA* plasmid (a gift from Michalis Averof; Addgene #102535) linearized with *NotI*. For *ΦC31*-mediated integrations, we used *ΦC31* integrase mRNA synthesized using plasmid *T7-ΦC31-nos3′UTR* (Addgene #182192) linearized with *EcoRI*. All mRNAs were synthesized using HiScribe T7 ARCA mRNA Kit (with tailing) (New England Biolabs #E2060S) and purified using Monarch RNA Cleanup Kit (New England Biolabs #T2040L), following manufacturer's instructions. mRNAs were stored as small aliquots at −70°C.

### Genomic DNA extraction

Cricket genomic DNA extractions were performed using the kit “NucleoSpin DNA Insect” (Macherey-Nagel #740470-50) following manufacturer's instructions with an extra step of 30 min incubation at 56°C after agitation at sample lysis stage. DNA samples were obtained either from 10 pooled embryos, 10 pooled first instar nymphs, single whole third instar nymphs, or single adult leg tips (tibia and tarsi).

### Mutagenesis of Gb-white using CRISPR/Cas9

Two sgRNAs targeting the *Gb-w* CDS were designed using the CRISPOR website (Concordet and Haeussler 2018). Guide sequences sgRNA#63 (5′-GAGTTCCTGGAAGAATTAAA-3′) and sgRNA#66 (5′-GAAGGTCGTGTTGCATATCT-3′) were selected based on their predicted efficiency (Moreno-Mateos *et al.* 2015). sgRNAs were synthesized using the EnGen sgRNA Synthesis Kit, *S. pyogenes* (New England BioLabs #E3322S) and purified using Monarch RNA Cleanup Kit (New England Biolabs #T2040L), following manufacturer's instructions. sgRNAs were stored as small aliquots at −70°C. EnGen Spy Cas9 NLS enzyme (New England BioLabs #M0646 M) (2.5 pmol/µl) and purified sgRNAs (2.5 pmol/µl each) were co-injected.

### Cricket imaging

Crickets were anesthetized with CO_2_ and immersed in 1× PBS, Triton 0.15% (PBS-T). Images were acquired with a stereo microscope (Stemi 2000-C, Carl Zeiss) equipped with a color camera (Invenio 20EIII, DeltaPix). Images taken at different focal planes were stacked using the Multifocus mode of the DeltaPix Insight imaging system. For fluorescence imaging, we used a Nikon SMZ18 stereomicroscope equipped with a DS-Ri2 color camera (Nikon) and the Nikon imaging system. Images were further processed with Affinity Designer 2 and Affinity Photo 2.

### EGFP-CenH3.1 imaging

Testes were dissected in PBS-T and fixed for 20 min at room temperature in 4% formaldehyde in PBS-T. They were then washed 3 times for 10 min in PBS-T and stained with DAPI (1 µg/ml) in mounting medium (Dako/Agilent). Nymphs were dissected in PBS-T and directly stained with DAPI without fixation. Images were acquired on a LSM800 confocal microscope with a 40× 1.4NA objective lens (Carl Zeiss), and images were processed with the Zen software (Carl Zeiss).

### Plasmid construction

PCR for cloning were all performed using Q5 High-Fidelity DNA Polymerase (New England Biolabs #M0491S). Oligonucleotide sequences are available in Supplementary Table 2. Assembly of vector and PCR products were made using NEBuilder HiFi DNA assembly kit (New England Biolabs #E5520S) or using T4 DNA ligase (New England Biolabs #B0202S). Assembled products were transformed into NEB 5-alpha Competent *E. coli* high efficiency cells (New England Biolabs #C2987H) by heat shock. Colonies were screened by PCR and final candidate plasmids were amplified and purified with the Nucleospin Plasmid kit (Macherey-Nagel #740588). Finally, all constructs were validated by full DNA sequencing (sequences are available in Supplementary File S1).

- *pBac-GbW-attB*. A 2,820 bp DNA fragment (in bold in Supplementary File S1) containing the *3xP3-DmHsp70* promoter, the *G. rubens/G. bimaculatus Gb-w* CDS, an *attB* site, and a multiple cloning site (MCS) was synthesized and cloned in the *pXL-BACII-LoxP-3xP3-DsRed-LoxP* plasmid (a gift from Christopher Potter; Addgene #26852) linearized with *XhoI* and *EcoRI*.
- *pMi-3xP3-DsRed-attP*. The *pMi-3xP3-DsRed* plasmid (a gift from Michalis Averof; Addgene #102539) was digested with *MluI* and *SpeI* to integrate a synthesized *attP* DNA cassette (Supplementary Table 2).
- *GbA3/4-hyPBase*. The *pUC19* plasmid was digested by *SphI* and *SmaI* enzymes. *Gb-actin3/4* promoter was amplified by PCR from genomic DNA using primers OMCL_121 and OMCL_122. *iHyPbase* coding sequence was amplified by PCR from *DmHsp70-iHyPBase* (Eckermann *et al*. 2018) using primers OMCL_123 and OMCL_124. *Gb-actin3/4* 3′UTR + 63 bp downstream was amplified by PCR from genomic DNA using primers OMCL_125 and OMCL_126. The 3 PCR products were assembled in digested *pUC19*.
- *pBac-GbW-attB{EGFP-CenH3.1}*. *Gb-CenH3.1* promoter and *CenH3.1* histone N-tail coding sequence were amplified by PCR from genomic DNA using primers OEG_122 and OEG_123. EGFP coding sequence was amplified by PCR using primers OEG_124 and OEG_125. *CenH3.1* histone fold coding sequence and *CenH3.1* 3′UTR + 1,883 bp downstream were amplified by PCR from genomic DNA using primers OEG_126 and OEG_127. The 3 PCR products were assembled in *pBac-GbW-attb* (digested with *EcoRV* and *XhoI*).

## Results

### Identification and mutagenesis of the *Gryllus white* gene

**Fig. 1.**
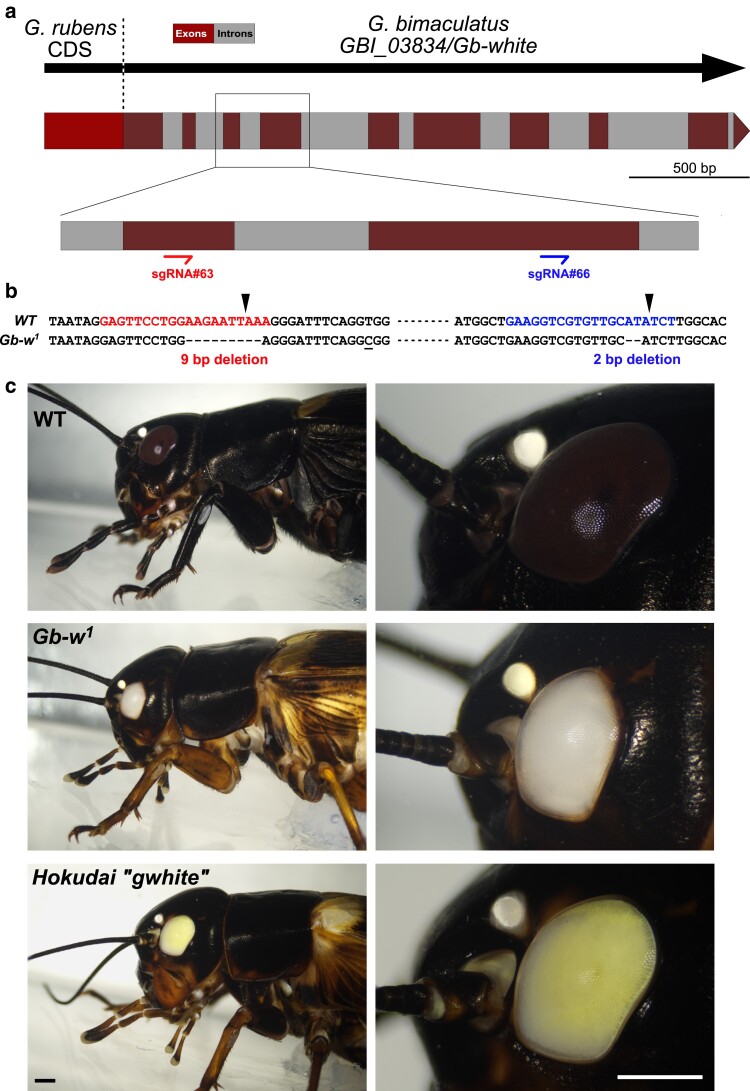
Identification and mutagenesis of the *G. bimaculatus white* gene. a) Scheme of the reconstructed *Gb-white* locus with predicted exons and introns. The missing 5′ part of the coding region is replaced with the corresponding *G. rubens* cDNA sequence. Inset shows position of the sgRNAs on target exons, whose corresponding protein sequence is indicated in Supplementary Fig. 1. b) DNA sequence alignments of sgRNA target regions in wild-type (WT) and *Gb-w^1^* mutant allele showing the deleted base pairs. The Cas9 cut sites are indicated (arrowheads). c) Images of WT, Gb-*w^1^*, and “Hokudai *gwhite*” adult crickets showing eye and ocelli. Bar: 1 mm.

The only currently available eye pigmentation mutant in *G. bimaculatus* is a recessive, autosomal mutation known as “Hokudai *gwhite*” (Niwa *et al.* 1997) but its molecular nature is unknown. Instead of the dark brown wild-type eye coloration, these mutant crickets have actually yellow eyes (see below). The “Hokudai *gwhite*” strain was chosen to sequence the *G. bimaculatus* genome (Ylla *et al.* 2021). A BLASTP search of *D. melanogaster* White protein against this reference genome sequence identifies GBI_03834 as the closest candidate ortholog. However, alignment of the predicted GBI_03834 protein with various insect White proteins suggests that it is incomplete, lacking its N-terminal region (Supplementary Fig. 1). In published transcriptomes of the closely related cricket *Gryllus rubens* (Berdan *et al.* 2016), we identified a transcript encoding a protein identical to GBI_03834 but with an extra 151 aa N-terminus (Supplementary Fig. 1). The DNA sequence corresponding to this N-terminus coding region is apparently absent in the *G. bimaculatus* reference genome, suggesting either sequencing or annotation errors, or the presence of a deletion at the *GBI_03834* locus. To directly test this candidate gene at the functional level, we generated mutant alleles using CRISPR/Cas9. We designed two single guide RNAs (sgRNAs) that target the available CDS of *GBI_03834* (Fig. 1a). These sgRNAs and Cas9 protein were injected in 209 eggs from a wild-type strain. All the surviving nymphs (*n* = 27) showed full or mosaic white eyes, confirming that *GBI_03834* is the *G. bimaculatus white* gene ortholog, that we named *Gb-white* (*Gb-w*). One of the injected crickets with full white eyes was backcrossed with a wild-type individual, and F1 animals were crossed together to generate homozygous mutant crickets. The selected mutant allele, *Gb-w^1^*, harbors two small deletions at both sgRNA targets, including a 2 bp deletion at the sgRNA#66 target that results in a frameshift (Fig. 1b). From our crossing experiments, we have observed that *Gb-w^1^* is a recessive, autosomal mutation. Homozygous *Gb-w^1^* crickets are viable and fertile but have pure white eyes, in contrast to the distinctive yellow eyes of “Hokudai *gwhite*” crickets (Fig. 1c). F1 crickets obtained from a cross between these two mutant strains have wild-type eye color (not shown), demonstrating that these mutations are not allelic.

### Detection of eye fluorescent proteins in the *Gb-w^1^* background

**Fig. 2.**
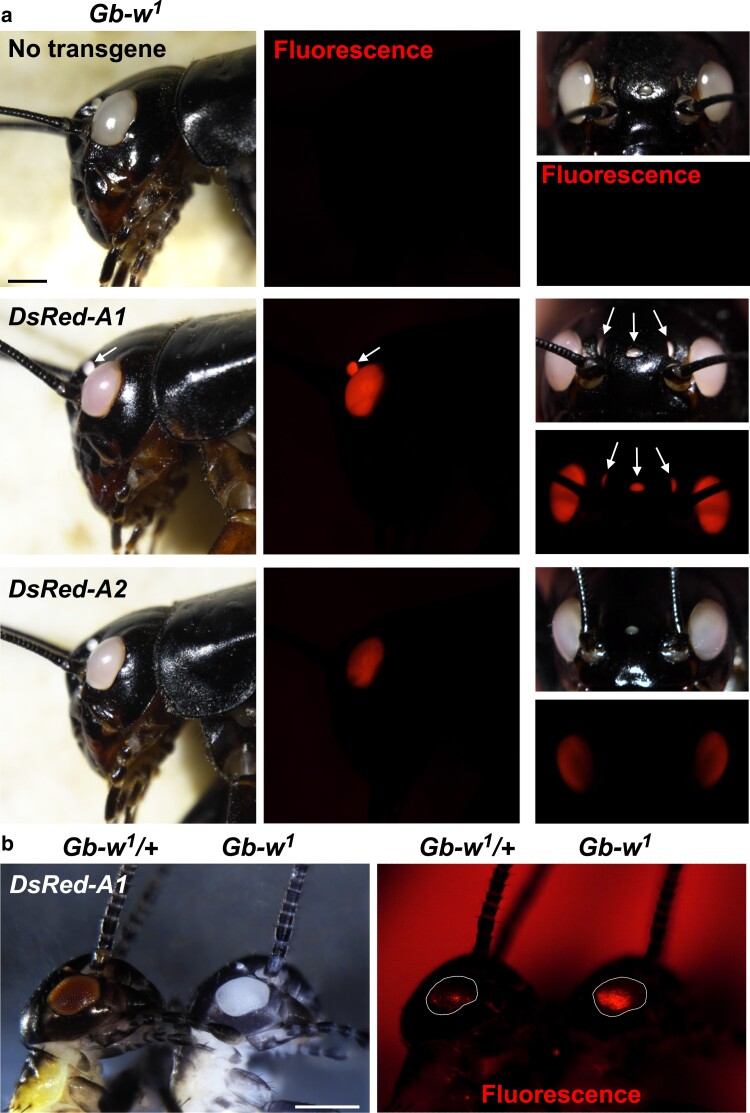
Detection of 3xP3-DsRed fluorescence in WT and Gb-*w^1^* backgrounds. a) Left panels: heads of *Gb-w^1^* adults with the indicated *pBac-3xP3-DsRed* insertion (heterozygous). Note the light pink eye coloration associated with the DsRed-A1 insertion. Middle panels: DsRed fluorescence detection from the same individuals. In the DsRed-A1 insertion, DsRed is detected in both the eyes and ocelli (arrows). In the DsRed-A2 insertion, fluorescence is weaker in the eye and not detected in ocelli. Right panels: frontal views (bright field and red fluorescence) of the same individuals. Bar: 1 mm. b) Left: bright field image of a heterozygote *Gb-w^1^*/+ and homozygote *Gb-w^1^* second instar nymphs with the same heterozygous DsRed-A1 insertion. Right: DsRed fluorescence is strongly quenched in the pigmented eye of the *Gb-w^1^*/+ nymph compared to the fluorescence detected in the unpigmented eye. Note that fluorescence does not cover the whole eye area (outlined) at this stage. Bar: 0.5mm.

Lack of eye pigmentation is advantageous for efficient detection of fluorescent proteins in the eyes with the 3xP3 artificial promoter. To assess the usefulness of our *Gb-w^1^* mutant line in this matter, we established transgenic cricket lines using *piggyBac*-mediated germline transformation using a vector containing the *3xP3-DsRed* marker (*pBac-3xP3-DsRed*). As a source of transposase, we first used a helper plasmid expressing hyperactive *piggyBac* transposase (hyPBase) under the control of the *D. melanogaster heat-shock protein 70* gene (*Dm-hsp70*) 5′ and 3′ regulatory sequences (Eckermann *et al.* 2018). Co-injection of *Dm-hyPBase* and *pBac-3xP3-DsRed* plasmids in *Gb-w^1^* eggs generated G0 adults with mosaic DsRed expression in eyes (34/36), indicating efficient integration in somatic cells. However, when crossed with *Gb-w^1^* individuals, none of the 34 fertile G0 individuals produced DsRed positive progeny (Supplementary Table 1). We then designed another helper plasmid (*GbA3/4-hyPBase*) that expresses hyPBase under the control of the cricket *GbA3/4* cytoplasmic actin promoter (Zhang *et al.* 2002). With this new helper plasmid, we detected DsRed in 73% of the surviving nymphs (*n* = 89) and one fertile adult among the 45 tested transmitted *3xP3-DsRed* to its progeny, suggesting a modest germline transformation efficiency with this helper plasmid (Supplementary Table 1). Nonetheless, several independent *pBac-3xP3-DsRed* insertions were recovered, as indicated by the different level of red fluorescence in the eye of G1 crickets. One of the isolated lines (DsRed-A1) showed robust red fluorescence in eyes and ocelli (Fig. 2a). Eyes from heterozygous DsRed-A1 crickets also display a visible pinkish coloration, indicating that DsRed can be detected under natural light in the *Gb-w^1^* background (Fig. 2a). We also selected another transgene insertion (DsRed-A2) showing moderate expression of DsRed in eyes but not in ocelli (Fig. 2a). As expected, when crossed with wild-type animals, we confirmed that DsRed fluorescence was strongly quenched in eyes with wild-type pigmentation, even in young nymphs where eye color is lighter than in adults (Fig. 2b). We also noticed that fluorescence from the DsRed-A1 transgene was not fully covering the whole eye area in young nymphs and was not detectable in ocelli (Fig. 2b), indicating that it can take several molts for the 3xP3-DsRed marker to achieve full expression. The *Gb-w^1^* mutant background is thus ideal for the screening of 3xP3-FP insertions in young nymphs. Note that our comparison of 3xP3-DsRed detection in *Gb-w^1^* and “Hokudai *gwhite*” nymphs did not reveal any significant difference, except that red fluorescence appeared more diffuse in the yellow eye mutant (Supplementary Fig. 2).

We then took advantage of our *Gb-w^1^* mutant line to test *Gryllus* transgenesis with a *Minos*-based vector marked with 3xP3-DsRed (*pMi-3xP3-DsRed*) (Pavlopoulos and Averof 2005). *Minos* is a DNA transposon isolated from *Drosophila hydei* which is used in invertebrate transgenesis applications (Franz and Savakis 1991; Pavlopoulos *et al.* 2004). Using an in vivo interplasmid transposition assay, it was previously shown that *Minos* can be active in *Gryllus* embryos in the presence of a helper plasmid encoding the *Minos* transposase (Zhang *et al.* 2002). However, no germline transformation was reported in this study. We thus chose to inject the *pMi-3xP3-DsRed* plasmid with in vitro synthesized mRNA encoding the *Minos* transposase in *Gb-w^1^* eggs. 86% (*n* = 29) of adult G0 crickets showed mosaic somatic expression of DsRed in eyes. In addition, 3 out of 17 fertile injected G0 adults transmitted *Mi-3xP3-DsRed* insertions to their progeny, demonstrating efficient germline transformation with this system (Supplementary Table 1). These lines have been maintained for 3 generations (and then discarded), thus suggesting that *pMi-3xP3-DsRed* insertions are stable.

### A mini-*white* marker for *Gryllus* transgenesis

**Fig. 3.**
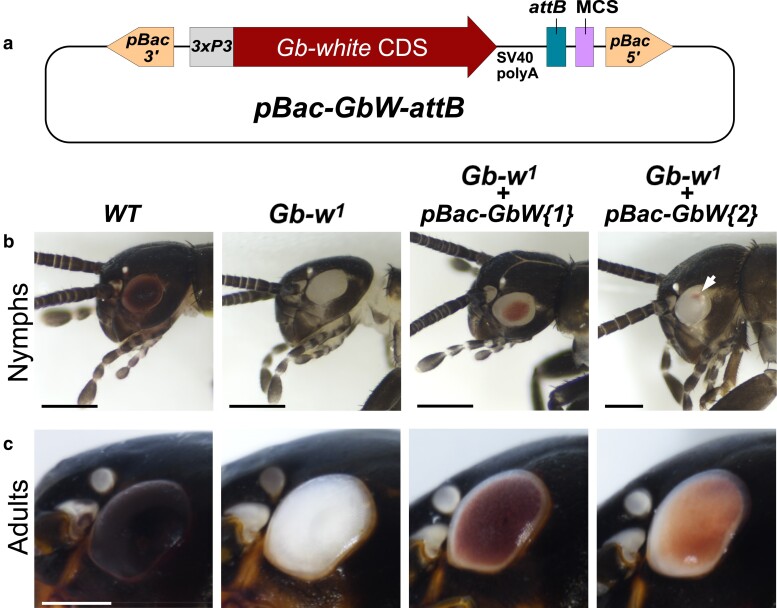
Detection of the *3xP3-white*^+^ transgenesis marker. a) Schematic representation of the *pBac-GbW-attB* transgenesis vector. Indicated elements are not drawn to scale. *pBac5′* and *pBac3′*, *piggyBac* 5′ and 3′ inverted terminal repeats. *3xP3*: *3xP3-DmHsp70* artificial promotor. *Gb-white* CDS: *G. rubens/G. bimaculatus* cDNA (see Fig. 1a). MCS, multiple cloning site. b) Images of control and transgenic nymphs with the indicated genotypes. Note the highly restricted *Gb-w^+^* expression associated with the *pBac-GbW-attB{2}* insertion (arrow). Bars: 0.5 mm. c) Images of control and transgenic adult eyes of the same genotypes as above. Bar: 1 mm.

We then sought to test the *Gb-white* gene as a potential transgenesis marker for *G. bimaculatus*. To minimize the size of this marker, we used a synthetic *Gb-w* CDS (without introns) under the control of *3xP3* and the basal *DmHsp70* promoter (*3xP3-DmHsp70*) to generate a *3xP3-Gb-w^+^* cassette. This cassette was inserted into a *piggyBac* vector along with a MCS and an *attB* integration sequence for potential use in *ΦC31* integrase-mediated transgenesis (Fig. 3a). This vector (*pBac-GbW-attB*) was co-injected with in vitro synthesized *hyPBase* mRNA in eggs from the *Gb-w^1^* strain. We observed that among the surviving G0 adults, 87% (*n* = 15) showed partial, mosaic eye pigmentation, demonstrating that our *3xP3-Gb-w^+^* marker is functional and providing definitive evidence that the mutant phenotype is due to mutation of the *Gb-white* gene. One of the fertile G0 individuals produced progeny with pigmented eye (Fig. 3b and Supplementary Table 1), indicating germline integration of *pBac-GbW-attB*. Interestingly, at least two independent insertions obtained from the same G0 founder showed distinct eye expression pattern. In first instar nymphs, one insertion was associated with a large central pigmented region of the eye whereas the other insertion expressed *Gb-w^+^* in a very small patch of ommatidia (Fig. 3b). Similar to what we observed for the *3xP3-DsRed* marker, expression of *3xP3-Gb-w^+^* eventually covered the whole eye at the adult stage, with the exception of a thin margin. Note that the intensity of adult eye coloration differed between the two insertions analyzed (Fig. 3c).

Finally, injection of *pBac-GbW-attB* with the *GbA3/4-hyPBase* helper plasmid in eggs from the “Hokudai *gwhite*” strain did not generate any *Gb-w^+^* mosaic (0%, *n* = 37), whereas the same plasmid mix injected in *Gb-w^1^* eggs generated a high frequency of mosaic nymphs (79%, *n* = 39). This result confirms that the yellow coloration of crickets from the “Hokudai *gwhite*” strain does not result from defective *Gb-w* expression.

### Transgenesis application: a fluorescent centromeric histone to mark cricket centromeres

**Fig. 4.**
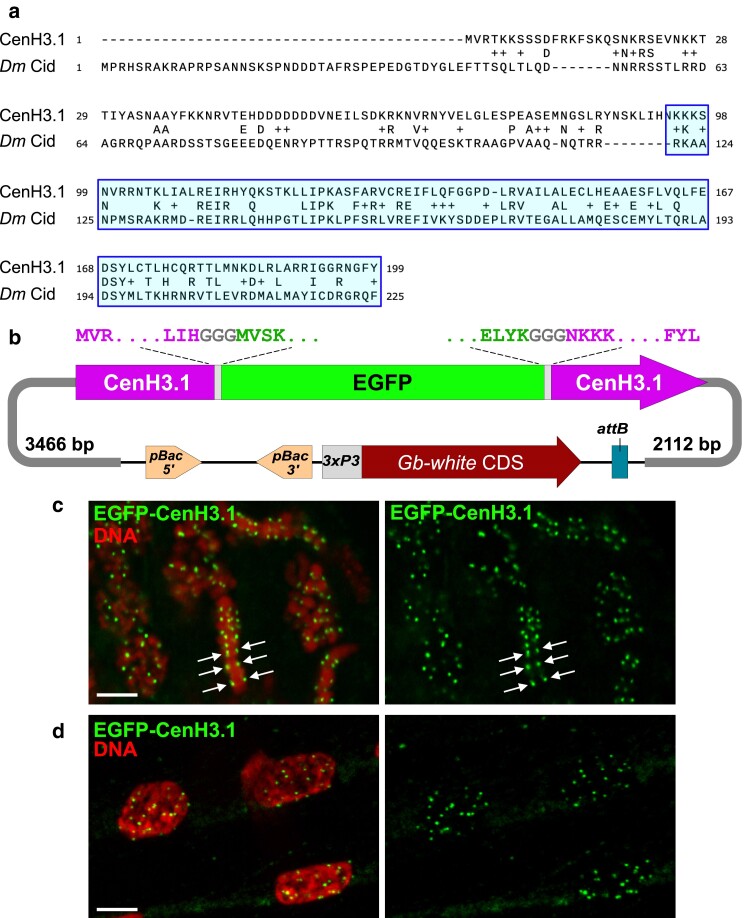
Generation of *pBac-GbW{EGFP-CenH3.1}* transgenic lines. a) Alignment of Cid and CenH3.1 proteins. The histone fold domain is highlighted in pale blue. b) Scheme of the *pBac-GbW{EGFP-CenH3.1}* transgene. The CenH3.1 coding sequence and amino-acids are in purple. EGFP is in green and linker peptides (GGG) in light gray. Genomic regions upstream the ATG codon and downstream the STOP codon, respectively, are represented by a thick dark gray line. Fragment sizes are indicated. c) EGFP-CenH3.1 in fixed G0 male germ cells stained for DNA (red). Arrows indicate individual centromeres on sister chromatids in metaphase. d) EGFP-CenH3.1 detection in cells from a G1 transgenic nymph. Bar: 5 µm.

To test our vector in a transgenesis application, we constructed a *pBac-GbW-attB* plasmid expressing a *G. bimaculatus* centromeric histone H3 (CenH3) candidate fused to EGFP. BLASTP search of the cricket genome with *D. melanogaster* CenH3/Cid identified GBI_16850 as a likely *Gryllus* CenH3 variant, which we named CenH3.1 (Fig. 4a). We cloned a single EGFP-CenH3.1 ORF under the control of the upstream and downstream endogenous genomic regions of CenH3.1. EGFP was inserted internally, between the N-terminus tail and the histone fold domain, following the same strategy used for *Drosophila* EGFP-Cid (Schuh *et al.* 2007) (Fig. 4b). After injection of this plasmid with *hyPBase* mRNA in *Gb-w^1^* eggs, 70 out of the 81 surviving adults (86%) displayed *Gb-w^+^* eye expression (Supplementary Table 1). Testicular follicles from 1 adult mosaic G0 male were dissected and fixed for examination in fluorescent microscopy. In some dividing premeiotic germ cell nuclei, EGFP-CenH3.1 was detected as bright foci on both sides of aligned metaphasic chromosomes (Fig. 4c), confirming successful expression and chromatin integration of the tagged centromeric histone. Progeny of 68 fertile G0 adults were screened for *Gb-w^+^* expression upon hatching. Transformants were recovered from 5 independent crosses (Supplementary Table 1), and we confirmed EGFP-CenH3.1 detection at centromeres in transgenic nymphs (Fig. 4d). Insertions were also confirmed by genomic DNA extraction and PCR analysis (Supplementary Fig. 3).

### Targeted transgenesis with the *ΦC31* integrase in *Gryllus*

**Fig. 5.**
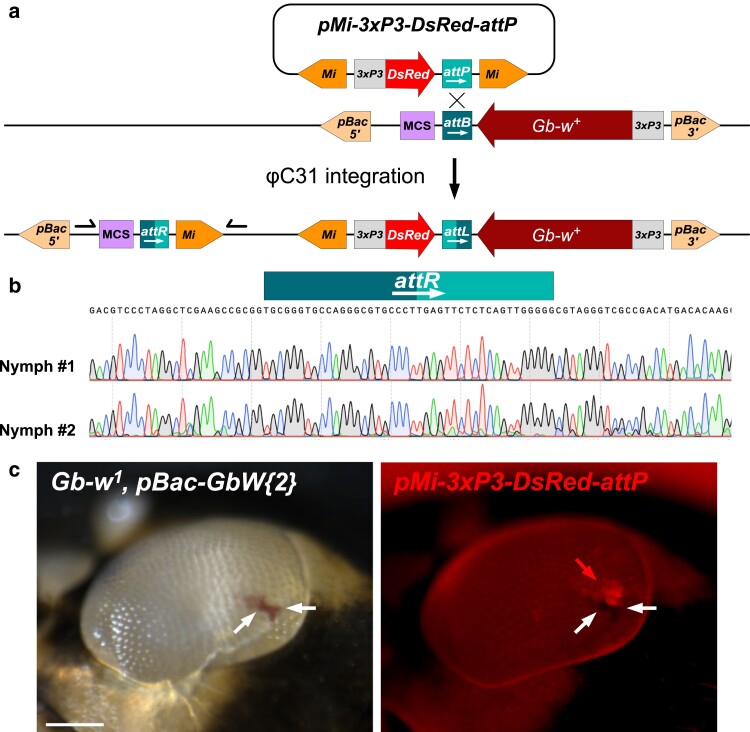
*ΦC31* integration. a) Scheme of the expected *ΦC31*-mediated integration of *pMI-3xP3-DsRed-attP* plasmid into the *pBac-GbW-attB* genomic insertion. b) Sanger electrophoregrams of *attR* region of 2 independent, randomly-selected integration events. Positions of PCR primers used to amplify this region are shown in a). The 2 sequences are identical, with their *attR* site indicated. c) A compound eye (anterior to the left) from a *Gb-w^1^*; *pBac-GbW-attB{2}/+* transgenic nymph with a *pMI-3xP3-DsRed-attP* integration event. Left: the restricted *Gb-w^+^* expression from the *pBac-GbW-attB{2}* insertion (see Fig. 3b) is indicated (white arrows). Right: DsRed fluorescence (red arrow) is detected at the edge of the *Gb-w^+^* region (which appears dark with fluorescent illumination). This suggests that expression of the DsRed fluorescence precedes eye pigmentation. Bar: 0.1 mm.

The *ΦC31* integrase system allows efficient integration of a single plasmid copy in a pre-existing genomic platform at single base resolution (Groth *et al.* 2004). This system is currently the most widely used transgenesis method in *D. melanogaster* (Bischof *et al.* 2007). *ΦC31*-mediated integration involves the specific and irreversible recombination of an *attB* sequence (usually on the donor plasmid) with and *attP* sequence (usually on the target chromosome). To test the *ΦC31* integrase system in *Gryllus*, we cloned an *attP* sequence into a *Minos* transgenesis vector (*pMi-3xP3-DsRed-attP*). Although this plasmid was designed to generate *attP* genomic platforms for future applications, we first tested it as a donor *attP* plasmid in combination with a previously generated *pBac-GbW-attB* transgenic insertion, which serves as a genomic *attB* target (Fig. 5a). *pMi-3xP3-DsRed-attP* plasmid DNA was co-injected with in vitro synthesized *ΦC31* integrase mRNA in eggs from a cross between heterozygous transgenic *pBac-GbW-attB* crickets in the *Gb-w^1^* genetic background. We scored injected *Gb-w^+^* nymphs for DsRed expression that should indicate *ΦC31*-mediated integration. As expected, in nymphs with a large *Gb-w^+^* expression domain, red fluorescence was strongly quenched by eye pigments, but we could occasionally detect it in the deep pseudopupil (not shown), as described for *Drosophila* (Berghammer *et al.* 1999). PCR and sequencing on these individual mosaic nymphs confirmed *pMi-3xP3-DsRed-attP* integration in the *pBac-GbW-attB* platform (Fig. 5b). In nymphs where the *pBac-GbW-attB* transgene is initially associated with a very small patch of pigmented ommatidia (see Fig. 3b), DsRed was detected at the edge of the *Gb-w^+^* area, suggesting that the appearance of DsRed fluorescence precedes pigmentation in these cells (Fig. 5c). These results indicate that the *ΦC31* system can be harnessed for targeted transgene insertion in *G. bimaculatus.*

## Discussion

The discovery of the first *Drosophila white* mutant allele by T.H. Morgan in 1910 played a pivotal role in the establishment of the chromosomal theory of Mendelian heredity (Morgan 1910). The *white* gene has since proven invaluable for modern *Drosophila* genetics, allowing for instance the discovery of position effect variegation (PEV) (Muller 1930; Green 2010; Elgin and Reuter 2013). Nonetheless, the *white* gene gained popularity in the *Drosophila* community primarily because of its effectiveness as a transgenesis marker for transposon-based germline transformation. To contribute to the development of a versatile transformation marker for crickets, we have generated a *Gb-w* mutant by CRISPR/Cas9. Our *Gb-w^1^* allele has been maintained as a homozygous stock for several generations in our laboratory. It is a healthy stock that does not show any overt phenotype besides the pure white eye coloration. In fact, several cricket strains with defective eye pigmentation have been successfully maintained in laboratories, including the “Hokudai *gwhite*” *G. bimaculatus* strain (Niwa *et al.* 1997) as well as spontaneous white and yellow eye mutants of the house cricket *Acheta domesticus* (Francikowski *et al.* 2019). In this latter species, recent CRISPR mutagenesis of the *Ad vermilion* gene resulted in lighter eye coloration with apparently no fitness cost, but eye pigmentation progressively increased during nymphal stages (Dossey *et al.* 2023). In contrast, *G. bimaculatus Gb-w^1^* mutant crickets display a stable, pure white eye coloration at all developmental stages.

We have designed a versatile transformation vector, *pBac-GbW-attB*, which contains the *3xP3-Gb-w^+^* marker, *piggyBac* terminal repeats as well as an *attB* sequence for *ΦC31*-mediated site-specific integration. Independent *piggyBac*-mediated insertions of this vector were associated with distinctive eye pigmentation patterns in newly hatched nymphs, likely reflecting the sensitivity of the *3xP3-Gb-w^+^* marker to its genomic environment and the progressive development of the nymphal cricket eye. This property of *3xP3-Gb-w^+^* is useful as it allows immediate identification of independent insertions during the screening of transgenic G1 nymphs, or the distinction of *Gb-w^+^* insertions at the adult stage. *3xP3-Gb-w^+^* is a relatively compact (2.7 kb) cassette that can be integrated in any transformation vector. It represents a convenient marker that does not necessitate a costly fluorescent light stereomicroscope. In addition, *Gb-w^+^* is not fluorescent and thus should not interfere with the expression of cargo genes tagged with a fluorescent protein. Finally, it can be detected simultaneously with *3xP3-FP* transgenes.

In this work, we also established *Minos*-mediated germline transformation in *G. bimaculatus*. A previous study had reported activity of *Minos* transposase expressed from a helper plasmid under the control of the *GbA3/4* promoter (Zhang *et al.* 2002). However, the authors observed that this promoter was active in extra-embryonic cells, such as vitellophages, and did not report germline transformation. Using mRNA as a source of *piggyBac* or *Minos* transposase, we were able to recover transformants from 7 to 17% of fertile G0 crickets, and founder animals frequently transmitted several independent insertions, as also observed in a previous report (Nakamura *et al.* 2010).

Finally, our study introduces the *ΦC31* integration system for site-specific transgene insertion in *Gryllus*. Our pilot experiment demonstrates precise integration of the *pMi-3xP3-DsRed-attP* plasmid into a single *attB* genomic target, thus paving the way for future site-specific transgenesis or recombinase-mediated cassette exchange (RMCE) (Bateman *et al.* 2006) applications in crickets.

In conclusion, we hope that these tools will encourage new researchers to adopt *G. bimaculatus* as a model insect for functional genetics.

## Supplementary Material

jkae235_Supplementary_Data

## Data Availability

The authors affirm that all data necessary for confirming the conclusions of the article are present within the article, figures, and tables. Strains and plasmids are available upon request. Supplemental material available at G3 online.
