# Supplementary material for: The white gene as a transgenesis marker for the cricket Gryllus bimaculatus
Source: G3 (Bethesda). 2024 Oct 15;14(12):jkae235. doi: 10.1093/g3journal/jkae235 (PMC11631507; doi:10.1093/g3journal/jkae235)
Supplement: jkae235_Supplementary_Data [file jkae235_supplementary_data.pdf]

Figure S1 – Alignment of White protein orthologs

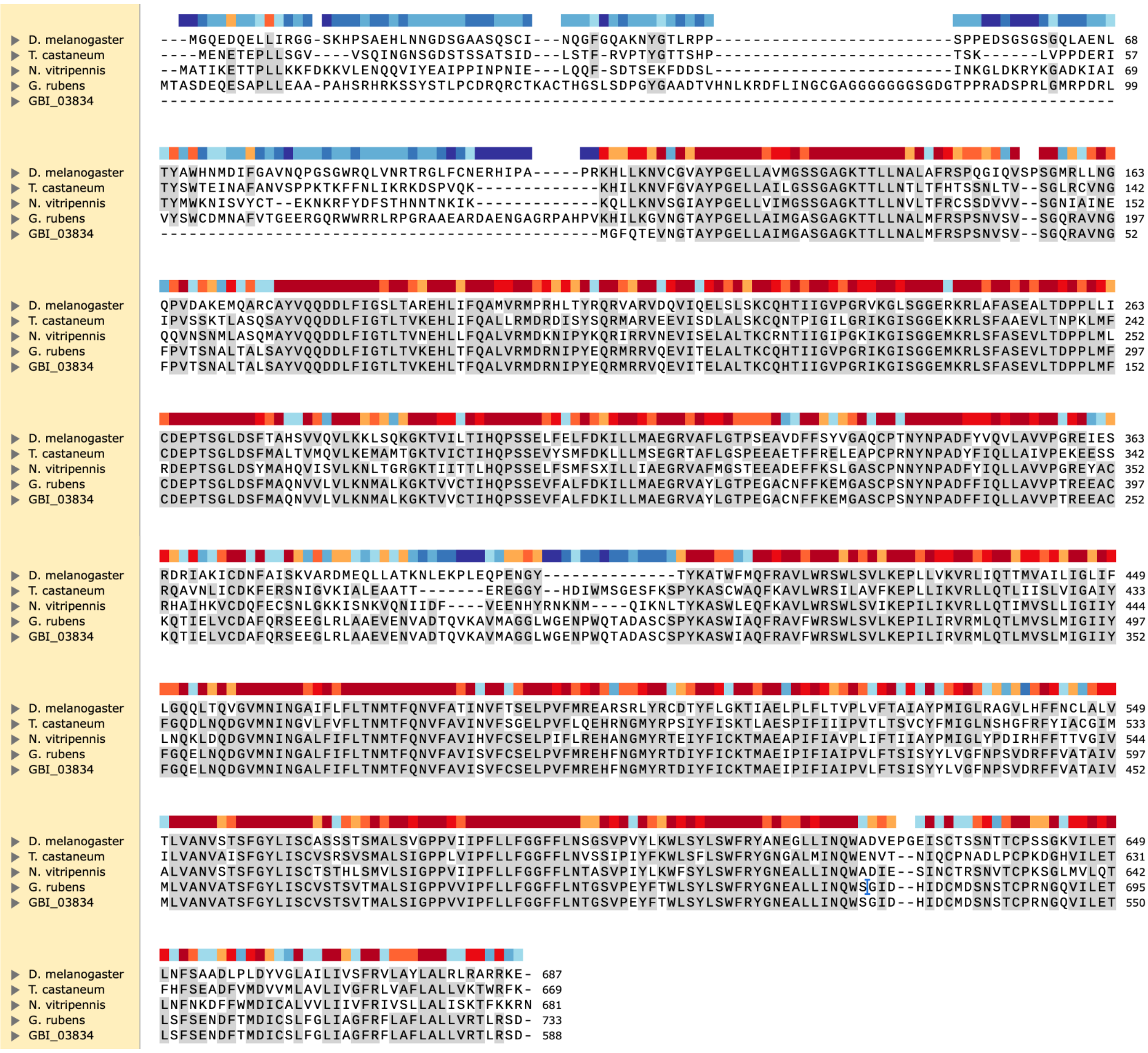

The light blue to dark red color scale indicates low to high residue conservation. The *Gryllus* White protein region corresponding to the two consecutive exons targeted by sgRNAs in **Figure 1a** are indicated by the red rectangles.

Figure S2 – Expression of 3xP3-DsRed in *WT* and mutant nymphs

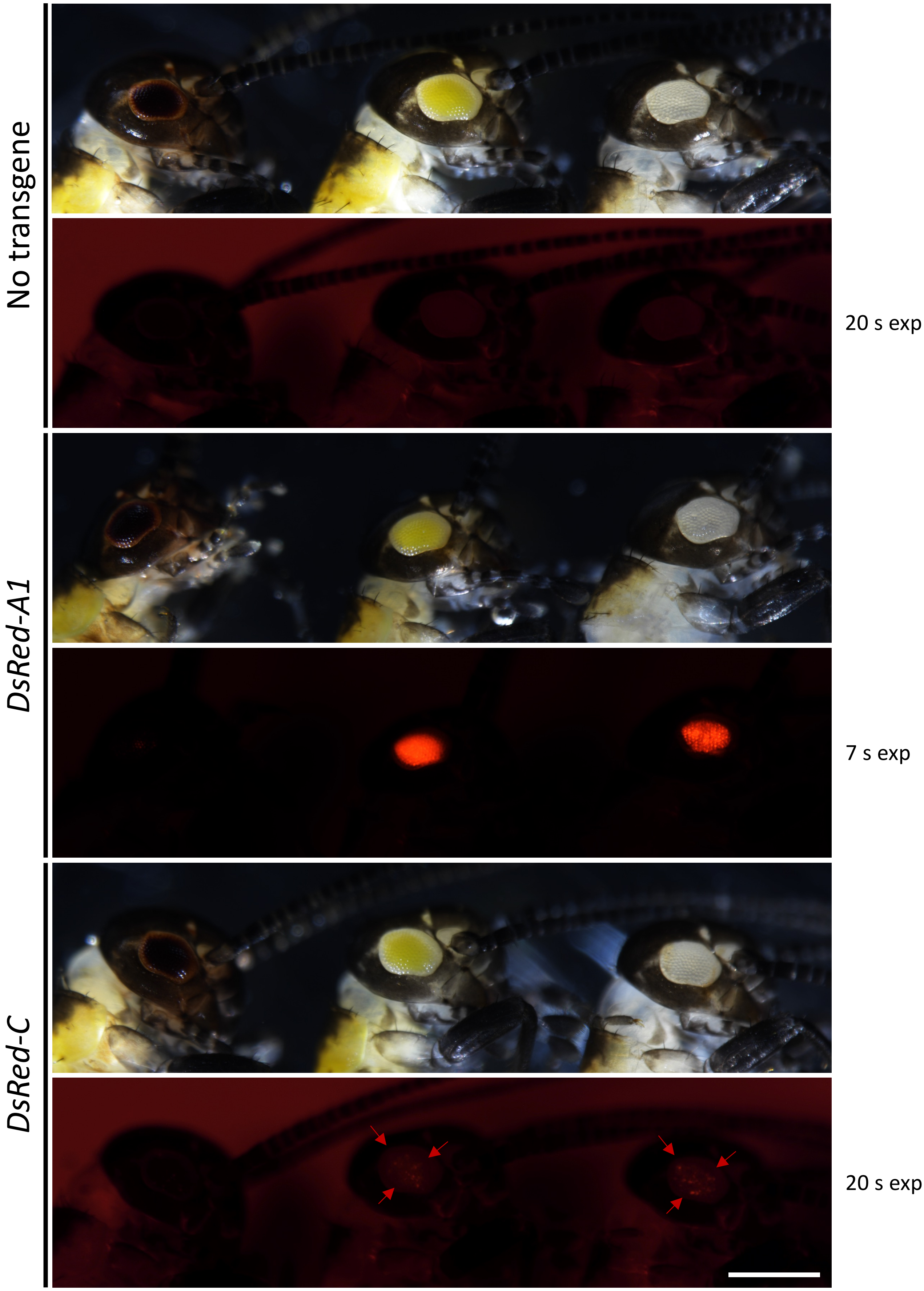

First instar WT (left), Hokudai *gwhite* (middle) or *Gb-w*[1] (right) nymphs with the indicated *pBac-3xP3-DsRed* transgene insertion. Expression of the DsRed-A1 insertion is detected in both mutant nymphs but appears more diffuse in yellow eyes. Expression of the DsRed-C insertion is only weakly detected in few ommatidia of Hokudai *gwhite* and *Gb-w*[1] eyes (red arrows). Bar: 0.5mm

Figure S3 – Example of *pBac-GbW-attB{EGFP-CenH3.1}* transgenic insertion validation by PCR analysis

(a)

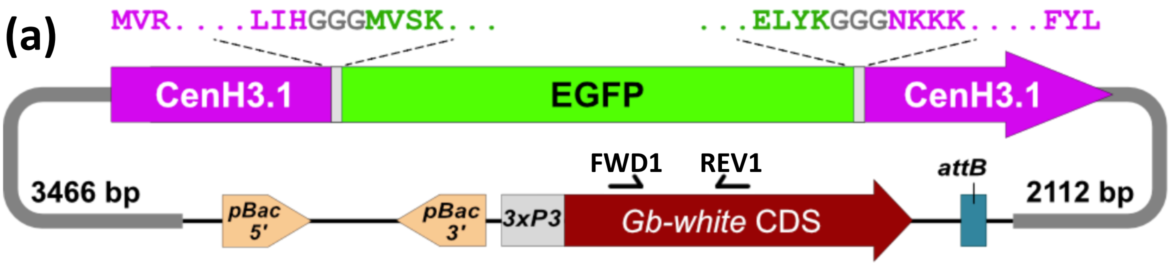

(b)

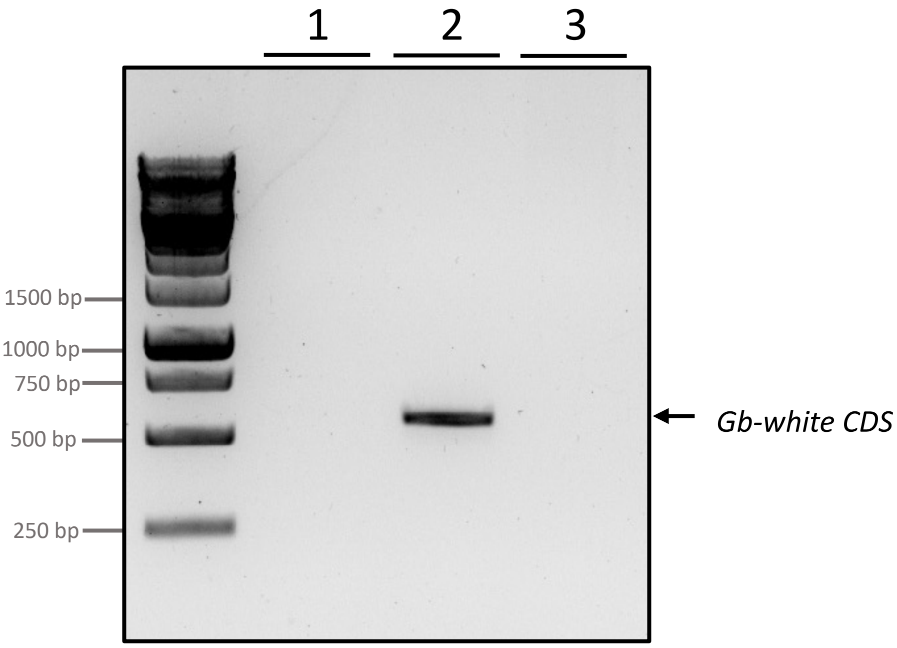

a. Scheme of the *pBac-GbW{EGFP-CenH3.1}* transgene. Positions of PCR primers used to amplify this region are shown: GBI_3834-FWD1 (FWD1) and GBI_3834-REV1 (REV1). The size of the expected amplicon is 553 bp.

b. PCR analysis of cricket genomic DNA. PCR were performed with primers GBI_3834-FWD1 and GBI_3834-REV1 using the following templates: DNA of a *Gb-w¹* adult cricket (1), DNA of a third-generation *pBac-GbW-attB{EGFP-CenH3.1}* transgenic adult cricket (2) and no DNA (3). PCR products were separated on a 1.5% agarose gel.

Table S1 – Summary of transgenesis experiments

Source of transposase	Donor plasmid	Injected embryos	Hatched nymphs	Hatching rate (%)	Adults		Offspring transmission / Fertile crossed adults	
DmHsp70-^{i}hyPBase (137 nM)	*pXL-BACII_LoxP-3xP3-DsRed-LoxP* (96 nM)	260	88	33.8	36	34	**0/34**	Mosaic eye
						2		Non mosaic eye
GbA3/4-^{i}hyPBase (106 nM)	*pXL-BACII_LoxP-3xP3-DsRed-LoxP* (96 nM)	271	89	32.8	61	59	**1/45**	Mosaic eye
						2		Non mosaic eye
GbA3/4-^{i}hyPBase (106 nM)	*pBac-GbW-attB* (119 nM)	166	77	46.4	24	24	**0/24**	Mosaic eye
						0		Non mosaic eye
mRNA ^{i}hyPBase (400 nM)	*pBac-GbW-attB* (131 nM)	81	30	37	15	13	**1/13**	Mosaic eye
						2		Non mosaic eye
mRNA minos transposase (483 nM)	*pMi(3xP3-DsRed)* (195 nM)	257	55	21.4	29	25	**3/17**	Mosaic eye
						4		Non mosaic eye
mRNA ^{i}hyPBase (400 nM)	*pBac-GbW-attB {EGFP-CenH3.1}* (65 nM)	437	142	32.5	81	70	**5/68**	Mosaic eye
						11		Non mosaic eye

Table S2 – Oligonucleotides

Cloning primers				
**Constructs**	**Primer name**	**Primer sequence 5'-3'**	**Assembled region**	**Plasmid linearization**
pMi-3xP3-DsRed-attP	*attP DNA synthesized cassette*	ctcatcaatgtatcttaaagcttatcgatacgcgtcccaggtcagaagcggttttcgggagtagt gccccaactggggtaacctttgagttctctcagttgggggcgtagggtcgccgacatgacaca aggggttactagttctagagcggccgccaccgcgggacgttac	N/A	MluI<br>NotI
GbA3/4-hyPBase	OMCL_121	aaacagctatgaccatgattacgccaagcttgcatgctgacgtcttctgacggttgtt	pUC19 + GbAct3/4(5’)	SphI
	OMCL_122	gaggatatgctcatcgtctaaagaactacccatggttagtagttgagtttacgggtactg	GbAct3/4(5’) + hyPBase	
	OMCL_123	tgcagtacccgtaaactcaactactaaccatgggtagttctttagacgatgagc	GbAct3/4(5’) + hyPBase	
	OMCL_124	gatttgaagatacaagggcaatgttcagaaacaactttggcacatatcaatattatg	hyPBase + GbAct3/4(3’)	
	OMCL_125	cataatattgatatgtgccaaagttgtttctgaacattgcccttgtatcttcaaatc	hyPBase + GbAct3/4(3’)	
	OMCL_126	aacgacggccagtgaattcgagctcggtacccgggaattggggaggaccaggaaataaagg	GbAct3/4(3’) + pUC19	KpnI
pBac-GbW-attB{EGFP-CenH3.1}	OEG_122	ttatatatatattttcttgttatagattaccccacacaccgaaggtg	pBac-GbW + CenH3.1(5’)	EcoRV
	OEG_123	tcctcgcccttgctcaccatgccgccgccatgaatcaattttgagttatagcgaagtg	CenH3.1(5’) + EGFP	
	OEG_124	atggtgagcaagggcgagga	CenH3.1(5’) + EGFP	
	OEG_125	cttgtacagctcgtccatgcc	EGFP + CenH3.1(3’)	
	OEG_126	ggcatggacgagctgtacaagggcggcggcaacaagaaaaaatccaatgtacgaagaa	EGFP + CenH3.1(3’)	
	OEG_127	ttcgagcctagggacgtctatgcggccgcctcgacattcccaaccttgaaccatacc	CenH3.1(3’) + pBac-GbW	XhoI

Genotyping primers			
**PCR Genotyping**	**Primer name**	**Primer sequence 5'-3'**	**Application**
Gb-w mutant screening	GBI_3834-FWD2	caggccttagttcgaatggacag	PCR
	GBI_3834-REV2	caagcttcctctcgagtggg	PCR
pBac-GbW{EGFP-CenH3.1} genotyping	GBI_3834-FWD1	gcgagctgtcaatggtttccc	PCR
	GBI_3834-REV1	gttactagggcaagatgcaccc	PCR
PhiC31 screening	OMCL111F	tcgccgcttggagctcccgtg	PCR
	M13	tgtaaaacgacggccagt	PCR
	T7	taatacgactcactataggg	Sequencing

File S1 – Plasmid DNA sequences

> *pBac-GbW-attB* (*GbW* cassette in bold)

GTGGCACTTTTCGGGGAAATGTGCGCGGAACCCCTATTTGTTTATTTTTCTAAATACATTCAAATATGTATCCGC
TCATGAGACAATAACCCTGATAAATGCTTCAATAATATTGAAAAAGGAAGAGTATGAGTATTCAACATTTCCGTG
TCGCCCTTATTCCCTTTTTTGCGGCATTTTGCCTTCCTGTTTTTGCTCACCCAGAAACGCTGGTGAAAGTAAAAG
ATGCTGAAGATCAGTTGGGTGCACGAGTGGGTTACATCGAACTGGATCTCAACAGCGGTAAGATCCTTGAGAGTT
TTCGCCCCGAAGAACGTTTTCCAATGATGAGCACTTTTAAAGTTCTGCTATGTGGCGCGGTATTATCCCGTATTG
ACGCCGGGCAAGAGCAACTCGGTCGCCGCATACACTATTCTCAGAATGACTTGGTTGAGTACTCACCAGTCACAG
AAAAGCATCTTACGGATGGCATGACAGTAAGAGAATTATGCAGTGCTGCCATAACCATGAGTGATAACACTGCGG
CCAACTTACTTCTGACAACGATCGGAGGACCGAAGGAGCTAACCGCTTTTTTGCACAACATGGGGGATCATGTAA
CTCGCCTTGATCGTTGGGAACCGGAGCTGAATGAAGCCATACCAAACGACGAGCGTGACACCACGATGCCTGTAG
CAATGGCAACAACGTTGCGCAAACTATTAACTGGCGAACTACTTACTCTAGCTTCCCGGCAACAATTAATAGACT
GGATGGAGGCGGATAAAGTTGCAGGACCACTTCTGCGCTCGGCCCTTCCGGCTGGCTGGTTTATTGCTGATAAAT
CTGGAGCCGGTGAGCGTGGGTCTCGCGGTATCATTGCAGCACTGGGGCCAGATGGTAAGCCCTCCCGTATCGTAG
TTATCTACACGACGGGGAGTCAGGCAACTATGGATGAACGAAATAGACAGATCGCTGAGATAGGTGCCTCACTGA
TTAAGCATTGGTAACTGTCAGACCAAGTTTACTCATATATACTTTAGATTGATTTAAAACTTCATTTTTAATTTA
AAAGGATCTAGGTGAAGATCCTTTTTGATAATCTCATGACCAAAATCCCTTAACGTGAGTTTTCGTTCCACTGAG
CGTCAGACCCCGTAGAAAAGATCAAAGGATCTTCTTGAGATCCTTTTTTTCTGCGCGTAATCTGCTGCTTGCAAA
CAAAAAAACCACCGCTACCAGCGGTGGTTTGTTTGCCGGATCAAGAGCTACCAACTCTTTTTCCGAAGGTAACTG
GCTTCAGCAGAGCGCAGATACCAAATACTGTCCTTCTAGTGTAGCCGTAGTTAGGCCACCACTTCAAGAACTCTG
TAGCACCGCCTACATACCTCGCTCTGCTAATCCTGTTACCAGTGGCTGCTGCCAGTGGCGATAAGTCGTGTCTTA
CCGGGTTGGACTCAAGACGATAGTTACCGGATAAGGCGCAGCGGTCGGGCTGAACGGGGGGTTCGTGCACACAGC
CCAGCTTGGAGCGAACGACCTACACCGAACTGAGATACCTACAGCGTGAGCTATGAGAAAGCGCCACGCTTCCCG
AAGGGAGAAAGGCGGACAGGTATCCGGTAAGCGGCAGGGTCGGAACAGGAGAGCGCACGAGGGAGCTTCCAGGGG
GAAACGCCTGGTATCTTTATAGTCCTGTCGGGTTTCGCCACCTCTGACTTGAGCGTCGATTTTTGTGATGCTCGT
CAGGGGGGCGGAGCCTATGGAAAAACGCCAGCAACGCGGCCTTTTTACGGTTCCTGGCCTTTTGCTGGCCTTTTG
CTCACATGTTCTTTCCTGCGTTATCCCCTGATTCTGTGGATAACCGTATTACCGCCTTTGAGTGAGCTGATACCG
CTCGCCGCAGCCGAACGACCGAGCGCAGCGAGTCAGTGAGCGAGGAAGCGGAAGAGCGCCCAATACGCAAACCGC
CTCTCCCCGCGCGTTGGCCGATTCATTAATGCAGCTGGCACGACAGGTTTCCCGACTGGAAAGCGGGCAGTGAGC
GCAACGCAATTAATGTGAGTTAGCTCACTCATTAGGCACCCCAGGCTTTACACTTTATGCTTCCGGCTCGTATGT
TGTGTGGAATTGTGAGCGGATAACAATTTCACACAGGAAACAGCTATGACCATGATTACGCCAAGCGCGCCCGCC
GGGTAACTCACGGGGTATCCATGTCCATTTCTGCGGCATCCAGCCAGGATACCCGTCCTCGCTGACGTAATATCC
CAGCGCCGCACCGCTGTCATTAATCTGCACACCGGCACGGCAGTTCCGGCTGTCGCCGGTATTGTTCGGGTTGCT
GATGCGCTTCGGGCTGACCATCCGGAACTGTGTCCGGAAAAGCCGCGACGAACTGGTATCCCAGGTGGCCTGAAC
GAACAGTTCACCGTTAAAGGCGTGCATGGCCACACCTTCCCGAATCATCATGGTAAACGTGCGTTTTCGCTCAAC
GTCAATGCAGCAGCAGTCATCCTCGGCAAACTCTTTCCATGCCGCTTCAACCTCGCGGGAAAAGGCACGGGCTTC
TTCCTCCCCGATGCCCAGATAGCGCCAGCTTGGGCGATGACTGAGCCGGAAAAAAGACCCGACGATATGATCCTG
ATGCAGCTAGATTAACCCTAGAAAGATAGTCTGCGTAAAATTGACGCATGCATTCTTGAAATATTGCTCTCTCTT
TCTAAATAGCGCGAATCCGTCGCTGTGCATTTAGGACATCTCAGTCGCCGCTTGGAGCTCCCGTGAGGCGTGCTT
GTCAATGCGGTAAGTGTCACTGATTTTGAACTATAACGACCGCGTGAGTCAAAATGACGCATGATTATCTTTTAC
GTGACTTTTAAGATTTAACTCATACGATAATTATATTGTTATTTCATGTTCTACTTACGTGATAACTTATTATAT
ATATATTTTCTTGTTATAGATATCAAGCTTATCGATACCGTCGAC**CTCGAGGCGGCCGCATAGACGTCCCTAGGC**
TCGAAGCCGCGGTGCGGGTGCCAGGGCGTGCCCTTGGGCTCCCCGGGCGCGTACTCCACCTCACCCATCTTAGTT
TGGACAAACCACAACTAGAATGCAGTGAAAAAAATGCTTTATTTGTGAAATTTGTGATGCTATTGCTTTATTTGT
AACCATTATAAGCTGCAATAAACAAGTTAACAACAACAATTGCATTCATTTTATGTTTCAGGTTCAGGGGGAGGT
GTGGGAGGTTTTTTAAAGCAAGTAAAACCTCTACAAATGTGGTATGGCTGATTATGATCTAGAGTCGCGGCCGTC
AATCTGAGCGAAGAGTTCTGACTAATAAAGCTAAGAATGCCAAGAATCTAAATCCAGCTATCAGTCCAAACAGGC
TGCAGATGTCCATAGTAAAATCATTCTCACTGAAGCTCAAGGTCTCTAGAATTACTTGACCATTTCGAGGACATG
TGCTGTTTGAGTCCATGCAATCAATATGATCAATACCAGACCACTGATTTATAAGTAACGCCTCATTGCCATAGC
GAAACCAAGATAAATAACTTAACCAAGTGAAATACTCAGGGACAGAACCAGTGTTCAGAAAAAATCCTCCAAATA
ATAAGAAAGGTATAACAACAGGAGGACCAATAGAGAGTGCCATTGTCACGCTGGTGCTGACACAAGATATGAGGT
AACCAAATGATGTTGCAACATTAGCCACCAACATAACTATAGCAGTTGCAACAAAAAATCGATCTACGGAAGGAT
TGAATCCTACTAAGTAATATGAAATAGATGTAAACAAAACTGGTATTGCTATGAATATAGGTATTTCTGCCATTG
TCTTGCAAATGAAGTATATATCTGTGCGATACATGCCATTGAAATGTTCTCGCATAAACACAGGTAGTTCACTAC
AAAAAACACTGATGACAGCAAAAACATTTTGAAATGTCATGTTTGTCAAAAATATGAAAAGAGCTCCGTTTATAT
TCATAACTCCATCTTGGTTGAGTTCTTGCCCAAAGTATATTATGCCAATCATTAATGATACCATCAGTGTCTGCA
GCATTCGTACTCGTATGAGGATAGGTTCTTTTAATACACTCAGCCAAGACCTCCAAAAAACAGCTCGGAATTGAG
CTATCCATGAAGCTTTGTATGGTGAACAACTGGCATCTGCTGTTTGCCAAGGATTTTCACCCCAGAGACCTCCAG
CCATTACAGCTTTTACCTGTGTATCAGCTACATTTTCAACTTCGGCAGCAAGGCGTAACCCTTCTTCTGATCGTT

GGAAAGCATCACAGACAAGTTCAATTGTCTGTTTACAAGCTTCCTCACGAGTGGGTACTACAGCTAACAATTGAA
TAAAAAAATCTGCTGGGTTGTAGTTACTAGGGCAAGATGCACCCATTTCTTTAAAAAAGTTACATGCACCTTCTG
GAGTGCCAAGATATGCAACACGACCTTCAGCCATGAGTAAAATTTTGTCAAATAGAGCAAATACTTCAGAAGATG
GTTGATGTATAGTACATACAACAGTTTTGCCCTTTAGGGCCATGTTTTTTAGTACAAGAACTACATTTTGAGCCA
TGAAAGAATCTAATCCAGATGTCGGCTCATCACAAAACATTAGGGGCGGATCTGTAAGAACTTCTGATGCAAAAG
AAAGACGTTTCATTTCTCCACCTGAAATCCCTTTAATTCTTCCAGGAACTCCTATTATAGTATGTTGACATTTTG
TGAGAGCTAATTCTGTGATAACTTCTTGGACACGTCTCATACGCTGTTCATACGGAATGTTTCTGTCCATTCGAA
CTAAGGCCTGAAATGTTAAGTGTTCCTTAACAGTCAATGTACCAATGAAGAGATCATCCTGTTGGACATATGCTG
ACAAAGCTGTTAGAGCATTGCTTGTAACAGGGAAACCATTGACAGCTCGCTGGCCACTTACAGAAACATTCGAAG
GAGATCGAAACATCAATGCATTTAGCAGGGTTGTTTTTCCTGCTCCACTAGCGCCCATAATAGCCAACAGTTCAC
CAGGATATGCTGTGCCATTTACTCCCTTGAGAATGTGCTTGACGGGGTGCGCGGGGCGGCCGGCGCCGTTCTCCG
CGTCGCGCGCCTCCGCCGCCCGTCCGGGCCGCAGGCGGCGCCACCAGCGCTGGCCGCGCTCCTCTCCGGTGACGA
AGGCGTTCATGTCGCACCACGAGTAGACGAGGCGGTCGGGCCGCATGCCCAGGCGCGGGGAATCGGCGCGAGGCG
GCGTGCCGTCCCCGCTGCCCCCGCCCCCACCGCCCCCGGCCCCGCAGCCGTTGATGAGGAAGTCTCGCTTGAGGT
TGTGCACGGTGTCCGCGGCGCCGTACCCGGGGTCCGACAGGGAGCCGTGGGTGCATGCTTTGGTGCAACGCTGCC
GGTCGCAAGGCAGAGTGCTGTAGGAGGACTTCCTGTGGCGGGAGTGCGCGGGCGCCGCCTCCAGCAGCGGGGCCG
ATTCCTGCTCGTCGCTAGCCGTCATGGTGGCGACCGGTGGATCCCGGGCCCGCGGTACCCCGATTGTTTAGCTTG
TTCAGCTGCGCTTGTTTATTTGCTTAGCTTTCGCTTAGCGACGTGTTCACTTTGCTTGTTTGAATTGAATTGTCG
CTCCGTAGACGAAGCGCCTCTATTTATACTCCGGCGGTCGAGGGTTCGAAATCGATAAGCTTGGATCCTAATTGA
ATTAGCTCTAATTGAATTAGTCTCTAATTGAATTAGATCCCCGGGCGAGCTCGAATTAACCATTGTGGGAACACT
AGAACTAGTGAATTCGATAAAAGTTTTGTTACTTTATAGAAGAAATTTTGAGTTTTTGTTTTTTTTAATAAATAA
ATAAACATAAATAAATTGTTTGTTGAATTTATTATTAGTATGTAAGTGTAAATATAATAAAACTTAATATCTATT
CAAATTAATAAATAAACCTCGATATACAGACCGATAAAACACATGCGTCAATTTTACACATGATTATCTTTAACG
TACGTCACAATATGATTATCTTTCTAGGGTTAATCTAGCTGCGTGTTCTGCAGCGTGTCGAGCATCTTCATCTGC
TCCATCACGCTGTAAAACACATTTGCACCGCGAGTCTGCCCGTCCTCCACGGGTTCAAAAACGTGAATGAACGAG
GCGCGCTCACTGGCCGTCGTTTTACAACGTCGTGACTGGGAAAACCCTGGCGTTACCCAACTTAATCGCCTTGCA
GCACATCCCCCTTTCGCCAGCTGGCGTAATAGCGAAGAGGCCCGCACCGATCGCCCTTCCCAACAGTTGCGCAGC
CTGAATGGCGAATGGGACGCGCCCTGTAGCGGCGCATTAAGCGCGGCGGGTGTGGTGGTTACGCGCAGCGTGACC
GCTACACTTGCCAGCGCCCTAGCGCCCGCTCCTTTCGCTTTCTTCCCTTCCTTTCTCGCCACGTTCGCCGGCTTT
CCCCGTCAAGCTCTAAATCGGGGGCTCCCTTTAGGGTTCCGATTTAGTGCTTTACGGCACCTCGACCCCAAAAAA
CTTGATTAGGGTGATGGTTCACGTAGTGGGCCATCGCCCTGATAGACGGTTTTTCGCCCTTTGACGTTGGAGTCC
ACGTTCTTTAATAGTGGACTCTTGTTCCAAACTGGAACAACACTCAACCCTATCTCGGTCTATTCTTTTGATTTA
TAAGGGATTTTGCCGATTTCGGCCTATTGGTTAAAAAATGAGCTGATTTAACAAAAATTTAACGCGAATTTTAAC
AAAATATTAACGCTTACAATTTAG

> *pMi-3xP3-DsRed-attP*

GTACCAAGTGCTTGAAATGCTAAATGTTTTCAATTTTTCGCCATTAAGACAAGCCTACACAAATGCTTCTATAAA
TTATGCCAAGCACGTTAGCAGCTTCTACGAGCCCCAACCACTATTAATTCGAACAGCATGTTTTTTTGCAGTGCG
CAATGTTTAACACACTATATTATCAATACTACTAAAGATAACACATACCAATGCATTTCGTCTCAAAGAGAATTT
TATTCTCTTCACGACGAAAAAAAAAGTTTTGCTCTATTTCCAACAACAACAAAAATATGAGTAATTTATTCAAAC
GGTTTGCTTAAGAGATAAGAAAAAAGTGACCACTATTAATTCGAACGCGGCGTAAGCTTACCTTAATCTCAAGAA
GAGCAAAACAAAAGCAACTAATGTAACGGAATCATTATCTAGTTATGATCTGCAAATAATGCTGCAGAGCTGGGG
GATCCCCGGCGGGCCCCCCCTCGAGGTCGACGGTATCGATAAGCTTGATATCGAATTCGAGCTCGCCCGGGGATC
TAATTCAATTAGAGACTAATTCAATTAGAGCTAATTCAATTAGGATCCAAGCTTATCGATTTCGAACCCTCGACC
GCCGGAGTATAAATAGAGGCGCTTCGTCTACGGAGCGACAATTCAATTCAAACAAGCAAAGTGAACACGTCGCTA
AGCGAAAGCTAAGCAAATAAACAAGCGCAGCTGAACAAGCTAAACAATCGGGGTACCGCTAGAGTCGACGGTACC
GCGGGCCCGGGATCCACCGGTCGCCACCATGGCCTCCTCCGAGGACGTCATCAAGGAGTTCATGCGCTTCAAGGT
GCGCATGGAGGGCTCCGTGAACGGCCACGAGTTCGAGATCGAGGGCGAGGGCGAGGGCCGCCCCTACGAGGGCAC
CCAGACCGCCAAGCTGAAGGTGACCAAGGGCGGCCCCCTGCCCTTCGCCTGGGACATCCTGTCCCCCCAGTTCCA
GTACGGCTCCAAGGTGTACGTGAAGCACCCCGCCGACATCCCCGACTACAAGAAGCTGTCCTTCCCCGAGGGCTT
CAAGTGGGAGCGCGTGATGAACTTCGAGGACGGCGGCGTGGTGACCGTGACCCAGGACTCCTCCCTGCAGGACGG
CTCCTTCATCTACAAGGTGAAGTTCATCGGCGTGAACTTCCCCTCCGACGGCCCCGTAATGCAGAAGAAGACTAT
GGGCTGGGAGGCCTCCACCGAGCGCCTGTACCCCCGCGACGGCGTGCTGAAGGGCGAGATCCACAAGGCCCTGAA
GCTGAAGGACGGCGGCCACTACCTGGTGGAGTTCAAGTCCATCTACATGGCCAAGAAGCCCGTGCAGCTGCCCGG
CTACTACTACGTGGACTCCAAGCTGGACATCACCTCCCACAACGAGGACTACACCATCGTGGAGCAGTACGAGCG
CGCCGAGGGCCGCCACCACCTGTTCCTGTAGCGGCCGCGACTCTAGATCATAATCAGCCATACCACATTTGTAGA
GGTTTTACTTGCTTTAAAAAACCTCCCACACCTCCCCCTGAACCTGAAACATAAAATGAATGCAATTGTTGTTGT
TAACTTGTTTATTGCAGCTTATAATGGTTACAAATAAAGCAATAGCATCACAAATTTCACAAATAAAGCATTTTT
TTCACTGCATTCTAGTTGTGGTTTGTCCAAACTCATCAATGTATCTTAAAGCTTATCGATACGCGTCCCAGGTCA

GAAGCGGTTTTCGGGAGTAGTGCCCCAACTGGGGTAACCTTTGAGTTCTCTCAGTTGGGGGCGTAGGGTCGCCGA
CATGACACAAGGGGTTACTAGTTCTAGAGCGGCCGCCACCGCGGGACGTTACACAATTCTAATATTAATTAAATT
ATTGTTTTAAGTATGATAGTAAATCACATTACGCCGCGTTCGAATTAATAGTGGTCACTTTTTTCTTATCTCTTA
AGCAAACCGTTTGAATAAATTACTCATATTTTTGTTGTTGTTGGAAATAGAGCAAAACTTTTTTTTTCGTCGTGA
AGAGAATAAAATTCTCTTTGAGACGAAATGCATTGGTATGTGTTATCTTTAGTAGTATTGATAATATAGTGTGTT
AAACATTGCGCACTGCAAAAAAAAACATGCTGTTCGAATTAATAGTGGTTGGGGCTCGTAGAAAACGAAAAATATC
TTAAGCTAGCATAGAGAATGGAGCAAAACTCAATTTGATGCGAGCTCCAATTCGCCCTATAGTGAGTCGTATTAC
AATTCACTGGCCGTCGTTTTACAACGTCGTGACTGGGAAAACCCTGGCGTTACCCAACTTAATCGCCTTGCAGCA
CATCCCCCTTTCGCCAGCTGGCGTAATAGCGAAGAGGCCCGCACCGATCGCCCTTCCCAACAGTTGCGCAGCCTG
AATGGCGAATGGACGCGCCCTGTAGCGGCGCATTAAGCGCGGCGGGTGTGGTGGTTACGCGCAGCGTGACCGCT
ACACTTGCCAGCGCCCTAGCGCCCGCTCCTTTCGCTTTCTTCCCTTCCTTTCTCGCCACGTTCGCCGGCTTTCCC
CGTCAAGCTCTAAATCGGGGGCTCCCTTTAGGGTTCCGATTTAGTGCTTTACGGCACCTCGACCCCAAAAAACTT
GATTAGGGTGATGGTTCACGTAGTGGGCCATCGCCCTGATAGACGGTTTTTCGCCCTTTGACGTTGGAGTCCACG
TTCTTTAATAGTGGACTCTTGTTCCAAACTGGAACAACACTCAACCCTATCTCGGTCTATTCTTTTGATTTATAA
GGGATTTTGCCGATTTCGGCCTATTGGTTAAAAAATGAGCTGATTTAACAAAAATTTAACGCGAATTTTAACAAA
ATATTAACGCTTACAATTTAGGTGGCACTTTTCGGGGAAATGTGCGCGGAACCCCTATTTGTTTATTTTTCTAAA
TACATTCAAATATGTATCCGCTCATGAGACAATAACCCTGATAAATGCTTCAATAATATTGAAAAAGGAAGAGTA
TGAGTATTCAACATTTCCGTGTCGCCCTTATTCCCTTTTTTGCGGCATTTTGCCTTCCTGTTTTTGCTCACCCAG
AAACGCTGGTGAAAGTAAAAGATGCTGAAGATCAGTTGGGTGCACGAGTGGGTTACATCGAACTGGATCTCAACA
GCGGTAAGATCCTTGAGAGTTTTCGCCCCGAAGAACGTTTTCCAATGATGAGCACTTTTAAAGTTCTGCTATGTG
GCGCGGTATTATCCCGTATTGACGCCGGGCAAGAGCAACTCGGTCGCCGCATACACTATTCTCAGAATGACTTGG
TTGAGTACTCACCAGTCACAGAAAAGCATCTTACGGATGGCATGACAGTAAGAGAATTATGCAGTGCTGCCATAA
CCATGAGTGATAACACTGCGGCCAACTTACTTCTGACAACGATCGGAGGACCGAAGGAGCTAACCGCTTTTTTGC
ACAACATGGGGGATCATGTAACTCGCCTTGATCGTTGGGAACCGGAGCTGAATGAAGCCATACCAAACGACGAGC
GTGACACCACGATGCCTGTAGCAATGGCAACAACGTTGCGCAAACTATTAACTGGCGAACTACTTACTCTAGCTT
CCCGGCAACAATTAATAGACTGGATGGAGGCGGATAAAGTTGCAGGACCACTTCTGCGCTCGGCCCTTCCGGCTG
GCTGGTTTATTGCTGATAAATCTGGAGCCGGTGAGCGTGGGTCTCGCGGTATCATTGCAGCACTGGGGCCAGATG
GTAAGCCCTCCCGTATCGTAGTTATCTACACGACGGGGAGTCAGGCAACTATGGATGAACGAAATAGACAGATCG
CTGAGATAGGTGCCTCACTGATTAAGCATTGGTAACTGTCAGACCAAGTTTACTCATATATACTTTAGATTGATT
TAAAACTTCATTTTTAATTTAAAAGGATCTAGGTGAAGATCCTTTTTGATAATCTCATGACCAAAATCCCTTAAC
GTGAGTTTTCGTTCCACTGAGCGTCAGACCCCGTAGAAAAGATCAAAGGATCTTCTTGAGATCCTTTTTTTCTGC
GCGTAATCTGCTGCTTGCAAACAAAAAAACCACCGCTACCAGCGGTGGTTTGTTTGCCGGATCAAGAGCTACCAA
CTCTTTTTCCGAAGGTAACTGGCTTCAGCAGAGCGCAGATACCAAATACTGTTCTTCTAGTGTAGCCGTAGTTAG
GCCACCACTTCAAGAACTCTGTAGCACCGCCTACATACCTCGCTCTGCTAATCCTGTTACCAGTGGCTGCTGCCA
GTGGCGATAAGTCGTGTCTTACCGGGTTGGACTCAAGACGATAGTTACCGGATAAGGCGCAGCGGTCGGGCTGAA
CGGGGGGTTCGTGCACACAGCCCAGCTTGGAGCGAACGACCTACACCGAACTGAGATACCTACAGCGTGAGCTAT
GAGAAAGCGCCACGCTTCCCGAAGGGAGAAAGGCGGACAGGTATCCGGTAAGCGGCAGGGTCGGAACAGGAGAGC
GCACGAGGGAGCTTCCAGGGGGAAACGCCTGGTATCTTTATAGTCCTGTCGGGTTTCGCCACCTCTGACTTGAGC
GTCGATTTTTGTGATGCTCGTCAGGGGGGCGGAGCCTATGGAAAAACGCCAGCAACGCGGCCTTTTTACGGTTCC
TGGCCTTTTGCTGGCCTTTTGCTCACATGTTCTTTCCTGCGTTATCCCCTGATTCTGTGGATAACCGTATTACCG
CCTTTGAGTGAGCTGATACCGCTCGCCGCAGCCGAACGACCGAGCGCAGCGAGTCAGTGAGCGAGGAAGCGGAAG
AGCGCCCAATACGCAAACCGCCTCTCCCCGCGCGTTGGCCGATTCATTAATGCAGCTGGCACGACAGGTTTCCCG
ACTGGAAAGCGGGCAGTGAGCGCAACGCAATTAATGTGAGTTAGCTCACTCATTAGGCACCCCAGGCTTTACACT
TTATGCTTCCGGCTCGTATGTTGTGTGGAATTGTGAGCGGATAACAATTTCACACAGGAAACAGCTATGACCATG
ATTACGCCAAGCTCGAAATTAACCCTCACTAAAGGGAACAAAAGCTG

> *GbA3/4-hyPBase*

GAGATACCTACAGCGTGAGCTATGAGAAAGCGCCACGCTTCCCGAAGGGAGAAAGGCGGACAGGTATCCGGTAAG
CGGCAGGGTCGGAACAGGAGAGCGCACGAGGGAGCTTCCAGGGGGAAACGCCTGGTATCTTTATAGTCCTGTCGG
GTTTCGCCACCTCTGACTTGAGCGTCGATTTTTGTGATGCTCGTCAGGGGGGCGGAGCCTATGGAAAAACGCCAG
CAACGCGGCCTTTTTACGGTTCCTGGCCTTTTGCTGGCCTTTTGCTCACATGTTCTTTCCTGCGTTATCCCCTGA
TTCTGTGGATAACCGTATTACCGCCTTTGAGTGAGCTGATACCGCTCGCCGCAGCCGAACGACCGAGCGCAGCGA
GTCAGTGAGCGAGGAAGCGGAAGAGCGCCCAATACGCAAACCGCCTCTCCCCGCGCGTTGGCCGATTCATTAATG
CAGCTGGCACGACAGGTTTCCCGACTGGAAAGCGGGCAGTGAGCGCAACGCAATTAATGTGAGTTAGCTCACTCA
TTAGGCACCCCAGGCTTTACACTTTATGCTTCCGGCTCGTATGTTGTGTGGAATTGTGAGCGGATAACAATTTCA
CACAGGAAACAGCTATGACCATGATTACGCCAAGCTTGCATGCTGACGTCTTCTGACGGTTGTTTATTACGAAAG
TGTTATTCAAACTACAATTCTTTAAATGAAAAAATTTATTTTTACTGCAATCTCGTGTAAAATAGTTGCAAGTAA
TACGTCCACATCTACGAATAATATATTATTTTTAATGATTTACATACAACAAATACATTAATAATCTCGTTAACA
GTGAACTGGACCGTTAACTTTCTCAGATTTCGTGGGCACATTCTGAAGGTCGAGGAATGCAGATAGTTGCGGTCT

TGGCCGTTACCTTTCTTCCACAAGCAGACAGCGTTTTCCAATGAGCTATTGAACATACGCATCAATCGGTGACGG
AATGCATGTTCCCAATGAAGCTTACGCACGTTTACTTATAGAACCCACATGATATCTGGTGGGATATGGTAGCCG
GCAATGTCAGTGAACTGCGTATTGATTGCGGAGGCCATGCCCTCAGACACGCCCTCTGGACGTGAGCAGAACGCA
CCCAGCTAGATTGTACCACGTGTCATTACCTTAAAACATTGAGAGGCTCTCCCGTATCAATGTAAATTAACGTTT
TCGGGTATTTTCCATTTTCGATGAGTTTCCATTAACGAGTCCTGCTATTGGTCTGCCGCATCTTCATGACTAGCG
GAAGTTTGAATTTTGAGGTTATGCGTTGTCGAATATTGGTTGACAACTCGACAACAATGCGTATTTAAGTGTTTT
ATCTAAATGTTCCACGAGTGTAGCATAAAAATCGCACTAATTAGCGAGTTGTCAAACAACGGAGCAAATAGACCC
CCGGTTCACTACAAAAAAATTACATCCTTATTTCTGGAATGAATGAAAAAAAATATTTCAAGAAAATTTTCAAGA
ATTGTAACAGTTTACATACATAATAAAATAGTATTAAACGGTCATAAGCTTTGAACTTCTTCTACCCGATGCATT
TCTCTGTGGAGAATCAAACCAGACTCTGTAGAGGGTCTTCAACCAATCAGAAATCTCCATTCGTTTTATGCCCAT
ATATGGCACGTACAAACCTGCTCGGATGGAGGGGGAAGTAGTGGACGAGTCCAAGTTGGCCAAAGCCACGGGGGA
GGCCACGGGATTTCTGGAGTACTACCATAGTATTTCATATACTTATATATTAAATTACGCTCTACTTTCAATTCA
AAGTTTAATTAATAAATAAAACAATAAAAAACTGAAATTATAGTTACCTTTTTTACCCATTTCCATGCTTAATTT
TTGATTAAAATGTATTGGGCGAGTCCATTACCTGAATAGACTTTCCCTGAACTGTTAGCTCGCTCACTTGCGCCA
AATTTGTTGTTGTGTGAGGACGTGCGTTGAAGGTAAGGAATCAAAGCTCAGCCAAGCGACATTATATTCTTTGTT
TTGTCTTTGCTATGCTATTATTACTGATTTTAAACGATGTTGGTTAGACATTTGGCGCGGCAAACTATGTATTAT
CAGCTGTTTAATGACATGCGGCAACCGGCGAATTTTCTGTCCTCATGGCAACCATGTTGAAATAGCATGTCTTGA
TATTCATATATTATTTTGTTATTTTTGTTTTAAACTGGCGAATGTAGCAATTGCTAGTGCGAGAATTCGAGGATC
CAGAAATATTAACAGGTTGTTGTGCGGTAGCAGGTGGTTCCCTGATCTATATATAGAATCTGTGCGAGGTTTTCG
GCTTCGAGCTTGTGCGCGCATTGAAACTTTTTGACAGTTCCTTCAAATGTTCAAAATAGTATTAGTTTTTGTTAC
GCCTAAATTTCTTGAGAATGTTGTTTAAACTCTTTTATTTTATGGCAAATGTTAAATTCATTGCTGTTTTTTTAG
CCGACACTTTTCGAAGCTTGTGTTTTGATATCTCGTTGTAAAGTAAATTCATTTATAAGTTCTCATTGACTTTTA
ACCGTTTTTAGCCGGCATGTTCCGGTAGGGAGCGTTGGTTCCACGCTGCTGGCTCGATACCATATATGGTAAAGA
AAGCTGCCATTGGATGAGAAGCATGCCCTTATTAGTTGGTGGTGGGGGTAGGGTAGGCATGGGAGGGGCAGGGCC
TCCGTAGTATATTACTCATAGGCCGGCTCCCGGCTTGAATCAGTCTGGTGCTAGTTCTCGTACTGTGAAGATAGC
AGTGTTGCGCTGCTGCGTTTTTGTATATATTACATTGTAAGTTTCTAGTGCTTCTCGAAGATACTTTGTGAATTT
GTGTTACGAGCATTTTGTGCCTTGTGGGATATCAGCACAATTTTTCGTGTGGATTTTCGCGTATGTGCTTGTGAG
CAGTTAGGGCGGGGCAACCGGCTTCGTCCACACTCCCAAGGATTTCGCCTTATTTGGCGGGTGTTTGCTTAGGGT
CTAGCCACTGAATATGGGCGGTGCCTGTGTAGTGGTTGCCGGTGTGGGGCTTAGTCTTGTCAATGGGCGGGAAAC
CGGTGTGGGGAAACTAGTAGCGAATTGGATGGTTAACTAGTTTACTCGTGCTACGGTTCTCTTAATTTTTTTTTT
ATTTCTTGCAGTACCCGTAAACTCAACTACTAACCATGGGTAGTTCTTTAGACGATGAGCATATCCTCTCTGCTC
TTCTGCAAAGCGATGACGAGCTTGTTGGTGAGGATTCTGACAGTGAAGTGTCAGATCACGTAAGTGAAGATGACG
TCCAGAGCGATACAGAAGAAGCGTTTATAGATGAGGTACATGAAGTGCAGCCAACGTCAAGCGGTAGTGAAATAT
TAGACGAACAAAATGTTATTGAACAACCAGGTTCTTCATTGGCTTCTAACAGAATCTTGACCTTGCCACAGAGGA
CTATTAGAGGTAAGAATAAACATTGTTGGTCAACTTCAAAGCCCACGAGGCGTAGCCGAGTCTCTGCACTGAACA
TTGTCAGATCTCAAAGAGGTCCGACGCGTATGTGCCGCAATATATATGACCCACTTTTATGCTTCAAACTATTTT
TTACTGATGAGATAATTTCGGAAATTGTAAAATGGACAAATGCTGAGATATCATTGAAACGTCGGGAATCTATGA
CAAGCGCTACATTTCGTGACACGAATGAAGATGAAATCTATGCTTTCTTTGGTATTCTGGTAATGACAGCAGTGA
GAAAAGATAACCACATGTCCACAGATGACCTCTTTGATCGATCTTTGTCAATGGTGTACGTCTCTGTAATGAGTC
GTGATCGTTTTGATTTTTTGATACGATGTCTTAGAATGGATGACAAAAGTATACGGCCCACACTTCGAGAAAACG
ATGTATTTACTCCTGTTAGAAAAATATGGGATCTCTTTATCCATCAGTGCATACAAAATTACACTCCAGGGGCTC
ATTTGACCATAGATGAACAGTTACTTGGTTTTAGAGGACGGTGTCCGTTTAGGGTGTATATCCCAAACAAGCCAA
GTAAGTATGGAATAAAAATCCTCATGATGTGTGACAGTGGTACGAAGTATATGATAAATGGAATGCCTTATTTGG
GAAGAGGAACACAGACCAACGGAGTACCACTCGGTGAATACTACGTGAAGGAGTTATCAAAGCCTGTGCACGGTA
GTTGTCGTAATATTACGTGTGACAATTGGTTCACCTCAATCCCTTTGGCAAAAAACTTACTACAAGAACCGTATA
AGTTAACCATTGTGGGAACCGTGCGATCAAACAAACGCGAGATACCGGAAGTACTGAAAAACAGTCGCTCCAGGC
CAGTGGGAACATCGATGTTTTGTTTTGACGGACCCCTTACTCTCGTCTCATATAAACCGAAGCCAGCTAAGATGG
TATACTTATTATCATCTTGTGATGAGGATGCTTCTATCAACGAAAGTACCGGTAAACCGCAAATGGTTATGTATT
ATAATCAAACTAAAGGCGGAGTGGACACGCTAGACCAAATGTGTTCTGTGATGACCTGCAGTAGGAAGACGAATA
GGTGGCCTATGGCATTATTGTACGGAATGATAAACATTGCCTGCATAAATTCTTTTATTATATACAGCCATAATG
TCAGTAGCAAGGGAGAAAAGGTTCAAAGTCGCAAAAAATTTATGAGAAACCTTTACATGGGCCTGACGTCATCGT
TTATGCGTAAGCGTTTAGAAGCTCCTACTTTGAAGAGATATTTGCGCGATAATATCTCTAATATTTTGCCAAAGG
AAGTGCCTGGTACATCAGATGACAGTACTGAAGAGCCAGTAATGAAAAAACGTACTTACTGTACTTACTGCCCCT
CTAAAATAAGGCGAAAGGCAAGCGCATCGTGCAAAAAATGCAAAAAAGTTATTTGTCGAGAGCATAATATTGATA
TGTGCCAAAGTTGTTTCTGAACATTGCCCTTGTATCTTCAAATCACTTTTTTCTATTGCTAGAAGTTTGCTACTT
CTATTTAAAGCTCCGCATGCCAGCTGGAGTCGGAAGAGACTTTTTGTCTTTTGTTCTGTGTAATTGATGAACAAT
TCTTAAGTCACCTGGTTGTATTATAAAAAAAATTTAGATTTGTGTTGAGTTAGACTGGGTTTCTCCAAGTATTCA
AAGGGTCTCTTTGATGAAAGGCCTCTTGATTATGTAAGGCATGCCATTTGAACAGTTGTAAGATATTTGGTAAAC
TTTTGTATCTGGAAAGTGTGTAATTTTTTGTAATACATTTTTCAGACTTTTTCCAATGTAAAAGTTGACAGTTTC
CTTTCATATTGGTTCTATTTTCAAGAACCTACCAAAGTGATCACACATGCACTAGGCCAAAGTATTAATTTAATC
CCATGTGCAAGACTTATCATTTAAAGATTAAATAAAGACATAAAATTGTAAATACTATGTTGTATACTTGTATGC

TTAATTAAATCCTCTACCTTTATTTCCTGGTCCTCCCCAATTCCCGGGTACCGAGCTCGAATTCACTGGCCGTCG
TTTTACAACGTCGTGACTGGGAAAACCCTGGCGTTACCCAACTTAATCGCCTTGCAGCACATCCCCCTTTCGCCA
GCTGGCGTAATAGCGAAGAGGCCCGCACCGATCGCCCTTCCCAACAGTTGCGCAGCCTGAATGGCGAATGGCGCC
TGATGCGGTATTTTCTCCTTACGCATCTGTGCGGTATTTCACACCGCATATGGTGCACTCTCAGTACAATCTGCT
CTGATGCCGCATAGTTAAGCCAGCCCCGACACCCGCCAACACCCGCTGACGCGCCCTGACGGGCTTGTCTGCTCC
CGGCATCCGCTTACAGACAAGCTGTGACCGTCTCCGGGAGCTGCATGTGTCAGAGGTTTTCACCGTCATCACCGA
AACGCGCGAGACGAAAGGGCCTCGTGATACGCCTATTTTTATAGGTTAATGTCATGATAATAATGGTTTCTTAGA
CGTCAGGTGGCACTTTTCGGGGAAATGTGCGCGGAACCCCTATTTGTTTATTTTTCTAAATACATTCAAATATGT
ATCCGCTCATGAGACAATAACCCTGATAAATGCTTCAATAATATTGAAAAAGGAAGAGTATGAGTATTCAACATT
TCCGTGTCGCCCTTATTCCCTTTTTTGCGGCATTTTGCCTTCCTGTTTTTGCTCACCCAGAAACGCTGGTGAAAG
TAAAAGATGCTGAAGATCAGTTGGGTGCACGAGTGGGTTACATCGAACTGGATCTCAACAGCGGTAAGATCCTTG
AGAGTTTTCGCCCCGAAGAACGTTTTCCAATGATGAGCACTTTTAAAGTTCTGCTATGTGGCGCGGTATTATCCC
GTATTGACGCCGGGCAAGAGCAACTCGGTCGCCGCATACACTATTCTCAGAATGACTTGGTTGAGTACTCACCAG
TCACAGAAAAGCATCTTACGGATGGCATGACAGTAAGAGAATTATGCAGTGCTGCCATAACCATGAGTGATAACA
CTGCGGCCAACTTACTTCTGACAACGATCGGAGGACCGAAGGAGCTAACCGCTTTTTTGCACAACATGGGGGATC
ATGTAACTCGCCTTGATCGTTGGGAACCGGAGCTGAATGAAGCCATACCAAACGACGAGCGTGACACCACGATGC
CTGTAGCAATGGCAACAACGTTGCGCAAACTATTAACTGGCGAACTACTTACTCTAGCTTCCCGGCAACAATTAA
TAGACTGGATGGAGGCGGATAAAGTTGCAGGACCACTTCTGCGCTCGGCCCTTCCGGCTGGCTGGTTTATTGCTG
ATAAATCTGGAGCCGGTGAGCGTGGGTCTCGCGGTATCATTGCAGCACTGGGGCCAGATGGTAAGCCCTCCCGTA
TCGTAGTTATCTACACGACGGGGAGTCAGGCAACTATGGATGAACGAAATAGACAGATCGCTGAGATAGGTGCCT
CACTGATTAAGCATTGGTAACTGTCAGACCAAGTTTACTCATATATACTTTAGATTGATTTAAAACTTCATTTTT
AATTTAAAAGGATCTAGGTGAAGATCCTTTTTGATAATCTCATGACCAAAATCCCTTAACGTGAGTTTTCGTTCC
ACTGAGCGTCAGACCCCGTAGAAAAGATCAAAGGATCTTCTTGAGATCCTTTTTTTCTGCGCGTAATCTGCTGCT
TGCAAACAAAAAAACCACCGCTACCAGCGGTGGTTTGTTTGCCGGATCAAGAGCTACCAACTCTTTTTCCGAAGG
TAACTGGCTTCAGCAGAGCGCAGATACCAAATACTGTTCTTCTAGTGTAGCCGTAGTTAGGCCACCACTTCAAGA
ACTCTGTAGCACCGCCTACATACCTCGCTCTGCTAATCCTGTTACCAGTGGCTGCTGCCAGTGGCGATAAGTCGT
GTCTTACCGGGTTGGACTCAAGACGATAGTTACCGGATAAGGCGCAGCGGTCGGGCTGAACGGGGGGTTCGTGCA
CACAGCCCAGCTTGGAGCGAACGACCTACACCGAACT

> *pBac-GbW-attB{EGFP-CenH3.1}*

GTGGCACTTTTCGGGGAAATGTGCGCGGAACCCCTATTTGTTTATTTTTCTAAATACATTCAAATATGTATCCGC
TCATGAGACAATAACCCTGATAAATGCTTCAATAATATTGAAAAAGGAAGAGTATGAGTATTCAACATTTCCGTG
TCGCCCTTATTCCCTTTTTTGCGGCATTTTGCCTTCCTGTTTTTGCTCACCCAGAAACGCTGGTGAAAGTAAAAG
ATGCTGAAGATCAGTTGGGTGCACGAGTGGGTTACATCGAACTGGATCTCAACAGCGGTAAGATCCTTGAGAGTT
TTCGCCCCGAAGAACGTTTTCCAATGATGAGCACTTTTAAAGTTCTGCTATGTGGCGCGGTATTATCCCGTATTG
ACGCCGGGCAAGAGCAACTCGGTCGCCGCATACACTATTCTCAGAATGACTTGGTTGAGTACTCACCAGTCACAG
AAAAGCATCTTACGGATGGCATGACAGTAAGAGAATTATGCAGTGCTGCCATAACCATGAGTGATAACACTGCGG
CCAACTTACTTCTGACAACGATCGGAGGACCGAAGGAGCTAACCGCTTTTTTGCACAACATGGGGGATCATGTAA
CTCGCCTTGATCGTTGGGAACCGGAGCTGAATGAAGCCATACCAAACGACGAGCGTGACACCACGATGCCTGTAG
CAATGGCAACAACGTTGCGCAAACTATTAACTGGCGAACTACTTACTCTAGCTTCCCGGCAACAATTAATAGACT
GGATGGAGGCGGATAAAGTTGCAGGACCACTTCTGCGCTCGGCCCTTCCGGCTGGCTGGTTTATTGCTGATAAAT
CTGGAGCCGGTGAGCGTGGGTCTCGCGGTATCATTGCAGCACTGGGGCCAGATGGTAAGCCCTCCCGTATCGTAG
TTATCTACACGACGGGGAGTCAGGCAACTATGGATGAACGAAATAGACAGATCGCTGAGATAGGTGCCTCACTGA
TTAAGCATTGGTAACTGTCAGACCAAGTTTACTCATATATACTTTAGATTGATTTAAAACTTCATTTTTAATTTA
AAAGGATCTAGGTGAAGATCCTTTTTGATAATCTCATGACCAAAATCCCTTAACGTGAGTTTTCGTTCCACTGAG
CGTCAGACCCCGTAGAAAAGATCAAAGGATCTTCTTGAGATCCTTTTTTTCTGCGCGTAATCTGCTGCTTGCAAA
CAAAAAAACCACCGCTACCAGCGGTGGTTTGTTTGCCGGATCAAGAGCTACCAACTCTTTTTCCGAAGGTAACTG
GCTTCAGCAGAGCGCAGATACCAAATACTGTTCTTCTAGTGTAGCCGTAGTTAGGCCACCACTTCAAGAACTCTG
TAGCACCGCCTACATACCTCGCTCTGCTAATCCTGTTACCAGTGGCTGCTGCCAGTGGCGATAAGTCGTGTCTTA
CCGGGTTGGACTCAAGACGATAGTTACCGGATAAGGCGCAGCGGTCGGGCTGAACGGGGGGTTCGTGCACACAGC
CCAGCTTGGAGCGAACGACCTACACCGAACTGAGATACCTACAGCGTGAGCTATGAGAAAGCGCCACGCTTCCCG
AAGGGAGAAAGGCGGACAGGTATCCGGTAAGCGGCAGGGTCGGAACAGGAGAGCGCACGAGGGAGCTTCCAGGGG
GAAACGCCTGGTATCTTTATAGTCCTGTCGGGTTTCGCCACCTCTGACTTGAGCGTCGATTTTTGTGATGCTCGT
CAGGGGGGCGGAGCCTATGGAAAAACGCCAGCAACGCGGCCTTTTTACGGTTCCTGGCCTTTTGCTGGCCTTTTG
CTCACATGTTCTTTCCTGCGTTATCCCCTGATTCTGTGGATAACCGTATTACCGCCTTTGAGTGAGCTGATACCG
CTCGCCGCAGCCGAACGACCGAGCGCAGCGAGTCAGTGAGCGAGGAAGCGGAAGAGCGCCCAATACGCAAACCGC
CTCTCCCCGCGCGTTGGCCGATTCATTAATGCAGCTGGCACGACAGGTTTCCCGACTGGAAAGCGGGCAGTGAGC
GCAACGCAATTAATGTGAGTTAGCTCACTCATTAGGCACCCCAGGCTTTACACTTTATGCTTCCGGCTCGTATGT
TGTGTGGAATTGTGAGCGGATAACAATTTCACACAGGAAACAGCTATGACCATGATTACGCCAAGCGCGCCCGCC

GGGTAACTCACGGGGTATCCATGTCCATTTCTGCGGCATCCAGCCAGGATACCCGTCCTCGCTGACGTAATATCC
CAGCGCCGCACCGCTGTCATTAATCTGCACACCGGCACGGCAGTTCCGGCTGTCGCCGGTATTGTTCGGGTTGCT
GATGCGCTTCGGGCTGACCATCCGGAACTGTGTCCGGAAAAGCCGCGACGAACTGGTATCCCAGGTGGCCTGAAC
GAACAGTTCACCGTTAAAGGCGTGCATGGCCACACCTTCCCGAATCATCATGGTAAACGTGCGTTTTCGCTCAAC
GTCAATGCAGCAGCAGTCATCCTCGGCAAACTCTTTCCATGCCGCTTCAACCTCGCGGGAAAAGGCACGGGCTTC
TTCCTCCCCGATGCCCAGATAGCGCCAGCTTGGGCGATGACTGAGCCGGAAAAAAGACCCGACGATATGATCCTG
ATGCAGCTAGATTAACCCTAGAAAGATAGTCTGCGTAAAATTGACGCATGCATTCTTGAAATATTGCTCTCTCTT
TCTAAATAGCGCGAATCCGTCGCTGTGCATTTAGGACATCTCAGTCGCCGCTTGGAGCTCCCGTGAGGCGTGCTT
GTCAATGCGGTAAGTGTCACTGATTTTGAACTATAACGACCGCGTGAGTCAAAATGACGCATGATTATCTTTTAC
GTGACTTTTAAGATTTAACTCATACGATAATTATATTGTTATTTCATGTTCTACTTACGTGATAACTTATTATAT
ATATATTTTCTTGTTATAGATTACCCCACACACCGAAGGTGCAGATTCATCTCCCAGAATGGCTTCTTGCACCAG
TCCAAGTACAAGGATCTGTAATCACCAAAAAAATAGTTTTTATTTACTATAATTTGCCCTCAATTGGCACTAAT
AACTGTATATTGAAAGGAACATACCATGAGAAACGTCCATAAAAACTTGAAGTGACTCTTCACATGGGTGAAAAA
GATCTGAAAATGGAGACAAACTTGAAATCTATCCCATCTTCAATAAATGCAACGAAGTAATAATTGAGATTCTTA
AAGATTATAGCAAATATTTACCTTTCACAAGGCTAGAATAAGCATCTACTGCACTTGCTGTAGCAGCATGTTCAA
CAGCACTAGAACCTGTATTAATCAACAACAAATTATTGCCAATACATTATGCTTTCAATAGCATGCTTCTTTTTG
CATTTTAGAAACATACAAGAGACTTACCATTTTCAGTTTGAAAAGCTGCCTGTGTACAAGGATCTGAAAATTAAA
AAATGCTGGTATCATGCTGGTATATTCTATTAAAACTATAAAAATATATTCTACTAAAACTATGTTCTAGTAATA
TTCTATTAAAAAAATCTTACCAATTACACCCATAGCTGCAGTGGGTGTCACCTCGCTGGGTCCAAACAAGGGATA
TGCAACTGAACATGAACATTAAAAAAATTCCACTATTTGTTACGCACCCATCATCTTCTCGAAGCCAAAATGCAC
GCTTCAGACTTCAAACAGCAGTAAATCACTTCATTTCTCTAATTCACTGACTGCGATATGGCGTTAACAGCCCTA
CAAACAGCACGTGAATATACAACCATAGGTCGACGAGGAAGGAATCCAATCCAACGGTAACCAACCGGCTCGTTT
GCGAAAAAATTTCAAAATGAAAAAGTAGTTCACCGATTCTCCGCTTGAGCGAGTTGGTGTTGTACAGATGTATAT
GGCGTCGCTCTGAACTCTGATTGGCTACTGGGCTACTGTATGGAATTTGAATAAGGACCGGATGTCACCCCACTG
TTAGAAATAGGAAATTTTTCGAGGGAAACTCGGAACTGGAAAATGGAGTAGGTAGCGTTAAATAGCAAGATAGTT
TTTATTGTCAGAATCTGTTTGACCGCAGCATGATACAATAAGAGTTTTTAACTGTTCAATTGTGAATGAAACAGT
TGAGTAGAAGTACTTTACAGTGGATTTGAAATGCAGAGAATTGGGTAAGGGCTTCAGTGGTATTTTATTGCAAGA
CTGAACATTAGTTTAAAAAATGTTAATTTGTGTTCATGTTGTTTGCATTTTTGAAGTAATTTTTATCTTGTTTCT
TTGTGAAGAAAGTTAATCTACCCAGCTTTCTGAACATTTTGAGACAATTCTTTGCATTTTAAGTAGTAATTTATA
ACTTACACCAGTTCATAGAGGAAATATTAATTATAGTAGTAATCAATATAGCGCGTATTTTCATTATTAGAGGAT
TGTCAGCAATGAAAAAATATATGCTCTAAATTTAATGAGTAAGTAAAGTATGAGGTTAGTGATAATATTTAAAAA
AATCACTTTTATTATTTCATCTTCAGAGTCAAAGAACCAAGCCAAAGCCCATGGACCACTGCAAGAAAGTGTTGT
TGAAGGCAAAGTGTTGTGATGCTGATGGTGGATCATAGATCATATCTCAACCCATCATAACAGGTGAAGATTGTT
CGTGTGATAATAGTGAAGAAGGCCAAGTGTTGTGATGACAACTGAATATCTTCATAACAGTGGTGAAGATTCTTT
GTTTTCTGAATAGCAATTGTGAATAAATCACACTGTAATTTACTAGTCAGGTCAAGTGGATATACTCTAATGCAT
TCCAAAATGAATCATATATTTCTTACAATATTTTTGCTTCCTTGCAATAAAACAATTTTAGCTAATTACTATACA
TTCCCTCTTCTTTAGCAAAGGAATCCAAACAAACAAAAGATTTAGATGATTAAAGTGCCATTCCCTTAATAGCAT
TAAAATATACAGTTGAAATTGTTTTATTAGTAAATAACATTAAGTAAAGCTTTGTTTATGTAATTGTGTGTTTAA
TATTAAGAAAATATATGCCAAAGTTAATCTTTTGTAGGGAAAAATAATATCCTATTGATGTGAACCACTATATTT
ACAGTACATTGTATACACTTATATTGAGCTGTCCATTAAATGTCCCTCCACAGTACTGCAATGAACTTAAAAAGG
CTCAGGGTGGGTTTTGATCTAGTGGAATTTGAAATGTAATGGGCGTCTTCACCTACATTTAAAAAAAAAATTATT
TTCCCTTTGCCCGAGAGAAGAAATATACGATTCTGAATATTTACATATATGTACATGTACTTAAAGACCAATAGA
AAAATAAAATTTTGCACAGCTTCAGCTGGCTAGCATTGTCCAATTTATACACAAGCAGTGAATGATCTTGTGATA
ATTTTTGTTTGTTAATTGCACAGTTCTAGTTTCTATATCTAAGGTTTAATATTTTTAGATAAAGGTAAATTTAAT
TTCATTTACTTTTTTGATACATTTACAAGAAATATTAAGAGACATTTACAACTTTTTTGGAACTTATTTCTTCCA
CTTGGGGAGGACATAGTTGTAGCTGGTAAATTGATGTTATTGGAAATTATTAAAGATTTTATTAAAGGTGGTTTT
TTGGGTAGCCTCTGGTAAATGTGTTTTTTTCAGAGCATGTATAGTGTGCTAAAAGATCAATTTTGAGTTCTAGTA
TGAAAAATGAGTTGATGCTAAATGATCTCTTAAATCTGTGGATTCTACAAAGAAAACAAAATACTTTCATCTCAC
CTCAACTACTACTGACATTAATTTATGATTAAGTGATTTGCTTATTGGAATAAAAGTAAAATTATATTCAGTAAT
CAGGCAAAAATTTGTGTTTGTCAGAGATGCTGAAAGTGATTTTATTCTGCTCATAGGTTTGAGCCACTCGGCATC
GTGATCTCGTGACACAACCTTCCCTTGCAAACAATCACTTGCAAAATAAAATGTTACTTTTACCACATATATGAT
ATAAAACAATTGTTATGGTTAGTGTAATAAAAAATGATAAAAAGGCTATGAAGATTGGTTACTCAAGTTCAATAT
TTCTATGGGTGTGTCAATAACACTTCGAGGAAATTACACACACACATGCTAAAATTCTCTATATTTTGTATGTTT
TACTTTCTCTTATACTGCAGATGATGATTGTGTACTCCATTCGTCAGTTTGTTCATTAGTAGTTGTCTTAGTTGC
AAGTGGCTTCAACATGATCTCTTGCAATGCTGTTGTCAAACAAGGCTTACTTGTTTCTCGACTTGTCAAACTGGT
CCCTTCTTACAGTAATTTTTCTATATCAACAATTTGACTGTAAATTAGTACTGGTTATTTATTATGTTCTACCGA
GGTATAACAAAAGAATTATTTGTTTTTTGATCTTGCAGGTGAAAATGGTTCGTACAAAGAAGTCTTCAAGTGATT
TCAGAAAATTTAGCAAACAGTCCAACAAGAGGTCTGAAGTAAATAAAAAGACAACAATCTATGCATCCAACGCTG
CTTATTTTAAGAAAAACCGTGTGACAGAACATGATGATGATGATGATGATGATGTGAATGAAATTTTATCAGACA
AGAGGAAAAATGTGCGAAATTATGTAGAATTAGGTTTAGAATCTCCAGAGGCCTCTGAAATGAATGGATCACTTC
GCTATAACTCAAAATTGATTCATGGCGGCGGCATGGTGAGCAAGGGCGAGGAGCTGTTCACCGGGGTGGTGCCCA

TCCTGGTCGAGCTGGACGGCGACGTAAACGGCCACAAGTTCAGCGTGTCCGGCGAGGGCGAGGGCGATGCCACCT
ACGGCAAGCTGACCCTGAAGTTCATCTGCACCACCGGCAAGCTGCCCGTGCCCTGGCCCACCCTCGTGACCACCC
TGACCTACGGCGTGCAGTGCTTCAGCCGCTACCCCGACCACATGAAGCAGCACGACTTCTTCAAGTCCGCCATGC
CCGAAGGCTACGTCCAGGAGCGCACCATCTTCTTCAAGGACGACGGCAACTACAAGACCCGCGCCGAGGTGAAGT
TCGAGGGCGACACCCTGGTGAACCGCATCGAGCTGAAGGGCATCGACTTCAAGGAGGACGGCAACATCCTGGGGC
ACAAGCTGGAGTACAACTACAACAGCCACAACGTCTATATCATGGCCGACAAGCAGAAGAACGGCATCAAGGTGA
ACTTCAAGATCCGCCACAACATCGAGGACGGCAGCGTGCAGCTCGCCGACCACTACCAGCAGAACACCCCCATCG
GCGACGGCCCCGTGCTGCTGCCCGACAACCACTACCTGAGCACCCAGTCCGCCCTGAGCAAAGACCCCAACGAGA
AGCGCGATCACATGGTCCTGCTGGAGTTCGTGACCGCCGCCGGGATCACTCTCGGCATGGACGAGCTGTACAAGG
GCGGCGGCAACAAGAAAAAATCCAATGTACGAAGAAACACTAAACTTATAGCTCTAAGAGAGATTCGACATTATC
AAAAAAGTACAAAATTATTAATACCCAAAGCCTCTTTTGCTAGAGTGTGTAGAGAAATTTTCCTGCAGTTTGGTG
GACCCGATTTAAGAGTTGCAATATTAGCTTTGGAATGCTTACATGAAGCTGCTGAATCATTCCTAGTTCAACTTT
TTGAAGATTCCTATTTGTGCACTTTACATTGTCAGAGGACGACATTAATGAATAAGGATCTTCGTTTAGCCCGCA
GGATAGGTGGAAGAAATGGATTTTATCTATAAATATCTGTCCTATGTTTTATGATGCTGTATTCAGTATTTTGTA
AGCTAGTACTAAACTCTATGTTATATCAGTGAACTTTATATACTCCTTTTTTGCAGTGATAAAATATGTATTTTC
ACAGGTTATAATTTCTAAATAAAAGATCGGTCATAAAAAGTCTGACAAAGAATTTTGTATGTGTTGCTTGCTTGC
AGTGTTCTGCATTGAGAGAGAGAGACAGAGACAGAGAGGGGGGGAGGAAGAGGGAGAGGGAGAAAGAGAGAGAGA
GAGAAATAAATAAATAAATGAATAATTGAGGTGTCCAGTGAAAGAGGGAGCTAACTCACAACTGAGTTTTGGGTT
AAATTTGTTTTATTACTAATTTATATAATAATGGGAATGTTTTTTCCTTGTTATCTTGAAGATAAACTTGTTTAA
TTGGGAAAATAAGATTGAGCTATTCTAGAAGTATTTTATAAATGTTACACAGTCCTGATTGATATCATGCTCTTT
TTTCCAAGTTGATTGATATATTATTTTTATTATTATTATTTCAACATTCTGTAAGTTTGACTATATTATTTTCTT
GGAGTGACACCACTAAGATATCTGTTCTATATGTTGCAAAGTCCTTGACTTCGAAAATTTCCAAAATTATAGCAT
GTTTTTCATCGTCTGTTAGTGTGTGGTGCTTGCAATTGTGATTAGACATATCAAGACATAATACACATTTGCACA
CTGGGCGTTGCTTGTCCTTGAGTACTGTTTTCCCTGTTGTGTCACTAACATTTTTCTTCACTGTTCCTCTTCCTG
CTACTTTCTTGTTTTGTTGCATCATAAATGTGTTCTTGCCACTTTTCTCCAAGCTGTCTGACACGAATCCATTTA
GTATTTTGTTTTTGGGCTCGTCAACCACCATTGTCATTAGCCCTGTAAGCATATATCCCTGATCCATATATAGAT
CTCATATAAGTATATCCCTGGAGGTTACGATGTTCCCCGTAAATCTGGCGCTGATCTACATGTCAGGTTTTGCCT
TCTGGTATCTCCACCAGCAGAGCACCATCTGAGCTGCTGACTCACCTCTTGATATAAGTAGTTGTAAGTAAATGT
GTGTGTGAGAGGAGAGGATGGGACGAAGAAGAGGAAGGAAAGATTGTGCGCTGTCCTTTGCCTGTTAAGGCCCTG
CTCTGGCATTCAACTGGTTGGAGAAACAACGAAAAACTACTGATCAATGTAGGCAGCTGCTAGAATTGTCCTCTA
GTCAGCGATTGAAAATGAAACTATCTAGCTAGTGTTGAAAATCCGTCAGGTCTCAAACCTTAGACCCTGCCTGTC
AGGGACACTGCAGTGGGAATAGTTGTCATGAGTCATCTCAGCCAATCACAGGGATCCTTTCTTTAACGAATAGCC
TTCATTCTTCATTGTGTTAATAATTGGTACAGATCGAAAACCTGGTGTAAGGAAGCCTGTGAAACTGAATATCGA
ATCGTTACTGGACATCAAATGTCTAATTTTCCTGTATTTATATTTTAAGGACTACTTGGAATGCTAGTAATGCAA
TTATTTGGTGTTTGAATTTGTGATGTCACACATACATTTGACGTTCTTGTACTATTGTTGGTGTTATGAACAACA
CATGTGAATGTGGCTTAGCTCTCTCTCGTGGAAATTATAGAATATAGGCAGGTCAACTATTTTAAAAGTTCAATA
TAATATCGGAATGGATTGTGATGCATTTATGAAATTTACAGAATAAATTGAAGGTGATTATAAGAGGATATGGAA
TGTGTCAAAAAGTCTATTTTTTACATTTTTGGGTTAGCTCCCTCTCTCATTGGACGCCTCCATTAATACCTTGTT
ATATTCAAGGAGGGAAAAAAATAAAAACTATTTTTGTAAAATAAAGCTGTTTAAAACGTGTGATTTGTGTAACGT
TTCTTAGTATATTGGGTTTAGAAAAACAAACAAAAATTGTTCTGTACTTTGCTAAGGCCTCAAACAAATTTTAGT
TTCAATTCAGTTTTAAATTTTTATAAGAACTCTCAATTTATGGTTTTTGTTAGCAACCTTTTGAATTATTCCAAA
GCAAAAATAAATAATACTAAAAGGTATGGTTCAAGGTTGGGAATGTCGAGGCGGCCGCATAGACGTCCCTAGGCT
CGAAGCCGCGGTGCGGGTGCCAGGGCGTGCCCTTGGGCTCCCCGGGCGCGTACTCCACCTCACCCATCTTAGTTT
GGACAAACCACAACTAGAATGCAGTGAAAAAAATGCTTTATTTGTGAAATTTGTGATGCTATTGCTTTATTTGTA
ACCATTATAAGCTGCAATAAACAAGTTAACAACAACAATTGCATTCATTTTATGTTTCAGGTTCAGGGGGAGGTG
TGGGAGGTTTTTTAAAGCAAGTAAAACCTCTACAAATGTGGTATGGCTGATTATGATCTAGAGTCGCGGCCGTCA
ATCTGAGCGAAGAGTTCTGACTAATAAAGCTAAGAATGCCAAGAATCTAAATCCAGCTATCAGTCCAAACAGGCT
GCAGATGTCCATAGTAAAATCATTCTCACTGAAGCTCAAGGTCTCTAGAATTACTTGACCATTTCGAGGACATGT
GCTGTTTGAGTCCATGCAATCAATATGATCAATACCAGACCACTGATTTATAAGTAACGCCTCATTGCCATAGCG
AAACCAAGATAAATAACTTAACCAAGTGAAATACTCAGGGACAGAACCAGTGTTCAGAAAAAATCCTCCAAATAA
TAAGAAAGGTATAACAACAGGAGGACCAATAGAGAGTGCCATTGTCACGCTGGTGCTGACACAAGATATGAGGTA
ACCAAATGATGTTGCAACATTAGCCACCAACATAACTATAGCAGTTGCAACAAAAAATCGATCTACGGAAGGATT
GAATCCTACTAAGTAATATGAAATAGATGTAAACAAAACTGGTATTGCTATGAATATAGGTATTTCTGCCATTGT
CTTGCAAATGAAGTATATATCTGTGCGATACATGCCATTGAAATGTTCTCGCATAAACACAGGTAGTTCACTACA
AAAAACACTGATGACAGCAAAAACATTTTGAAATGTCATGTTTGTCAAAAATATGAAAAGAGCTCCGTTTATATT
CATAACTCCATCTTGGTTGAGTTCTTGCCCAAAGTATATTATGCCAATCATTAATGATACCATCAGTGTCTGCAG
CATTCGTACTCGTATGAGGATAGGTTCTTTTAATACACTCAGCCAAGACCTCCAAAAAACAGCTCGGAATTGAGC
TATCCATGAAGCTTTGTATGGTGAACAACTGGCATCTGCTGTTTGCCAAGGATTTTCACCCCAGAGACCTCCAGC
CATTACAGCTTTTACCTGTGTATCAGCTACATTTTCAACTTCGGCAGCAAGGCGTAACCCTTCTTCTGATCGTTG
GAAAGCATCACAGACAAGTTCAATTGTCTGTTTACAAGCTTCCTCACGAGTGGGTACTACAGCTAACAATTGAAT
AAAAAAATCTGCTGGGTTGTAGTTACTAGGGCAAGATGCACCCATTTCTTTAAAAAAGTTACATGCACCTTCTGG

AGTGCCAAGATATGCAACACGACCTTCAGCCATGAGTAAAATTTTGTCAAATAGAGCAAATACTTCAGAAGATGG
TTGATGTATAGTACATACAACAGTTTTGCCCTTTAGGGCCATGTTTTTTAGTACAAGAACTACATTTTGAGCCAT
GAAAGAATCTAATCCAGATGTCGGCTCATCACAAAACATTAGGGGCGGATCTGTAAGAACTTCTGATGCAAAAGA
AAGACGTTTCATTTCTCCACCTGAAATCCCTTTAATTCTTCCAGGAACTCCTATTATAGTATGTTGACATTTTGT
GAGAGCTAATTCTGTGATAACTTCTTGGACACGTCTCATACGCTGTTCATACGGAATGTTTCTGTCCATTCGAAC
TAAGGCCTGAAATGTTAAGTGTTCCTTAACAGTCAATGTACCAATGAAGAGATCATCCTGTTGGACATATGCTGA
CAAAGCTGTTAGAGCATTGCTTGTAACAGGGAAACCATTGACAGCTCGCTGGCCACTTACAGAAACATTCGAAGG
AGATCGAAACATCAATGCATTTAGCAGGGTTGTTTTTCCTGCTCCACTAGCGCCCATAATAGCCAACAGTTCACC
AGGATATGCTGTGCCATTTACTCCCTTGAGAATGTGCTTGACGGGGTGCGCGGGGCGGCCGGCGCCGTTCTCCGC
GTCGCGCGCCTCCGCCGCCCGTCCGGGCCGCAGGCGGCGCCACCAGCGCTGGCCGCGCTCCTCTCCGGTGACGAA
GGCGTTCATGTCGCACCACGAGTAGACGAGGCGGTCGGGCCGCATGCCCAGGCGCGGGGAATCGGCGCGAGGCGG
CGTGCCGTCCCCGCTGCCCCCGCCCCCACCGCCCCCGGCCCCGCAGCCGTTGATGAGGAAGTCTCGCTTGAGGTT
GTGCACGGTGTCCGCGGCGCCGTACCCGGGGTCCGACAGGGAGCCGTGGGTGCATGCTTTGGTGCAACGCTGCCG
GTCGCAAGGCAGAGTGCTGTAGGAGGACTTCCTGTGGCGGGAGTGCGCGGGCGCCGCCTCCAGCAGCGGGGCCGA
TTCCTGCTCGTCGCTAGCCGTCATGGTGGCGACCGGTGGATCCCGGGCCCGCGGTACCCCGATTGTTTAGCTTGT
TCAGCTGCGCTTGTTTATTTGCTTAGCTTTCGCTTAGCGACGTGTTCACTTTGCTTGTTTGAATTGAATTGTCGC
TCCGTAGACGAAGCGCCTCTATTTATACTCCGGCGGTCGAGGGTTCGAAATCGATAAGCTTGGATCCTAATTGAA
TTAGCTCTAATTGAATTAGTCTCTAATTGAATTAGATCCCCGGGCGAGCTCGAATTAACCATTGTGGGAACACTA
GAACTAGTGAATTCGATAAAAGTTTTGTTACTTTATAGAAGAAATTTTGAGTTTTTGTTTTTTTTTAATAAATAAA
TAAACATAAATAAATTGTTTGTTGAATTTATTATTAGTATGTAAGTGTAAATATAATAAAACTTAATATCTATTC
AAATTAATAAATAAACCTCGATATACAGACCGATAAAACACATGCGTCAATTTTACACATGATTATCTTTAACGT
ACGTCACAATATGATTATCTTTCTAGGGTTAATCTAGCTGCGTGTTCTGCAGCGTGTCGAGCATCTTCATCTGCT
CCATCACGCTGTAAAACACATTTGCACCGCGAGTCTGCCCGTCCTCCACGGGTTCAAAAACGTGAATGAACGAGG
CGCGCTCACTGGCCGTCGTTTTACAACGTCGTGACTGGGAAAACCCTGGCGTTACCCAACTTAATCGCCTTGCAG
CACATCCCCCTTTCGCCAGCTGGCGTAATAGCGAAGAGGCCCGCACCGATCGCCCTTCCCAACAGTTGCGCAGCC
TGAATGGCGAATGGGACGCGCCCTGTAGCGGCGCATTAAGCGCGGCGGGTGTGGTGGTTACGCGCAGCGTGACCG
CTACACTTGCCAGCGCCCTAGCGCCCGCTCCTTTCGCTTTCTTCCCTTCCTTTCTCGCCACGTTCGCCGGCTTTC
CCCGTCAAGCTCTAAATCGGGGGCTCCCTTTAGGGTTCCGATTTAGTGCTTTACGGCACCTCGACCCCAAAAAAC
TTGATTAGGGTGATGGTTCACGTAGTGGGCCATCGCCCTGATAGACGGTTTTTCGCCCTTTGACGTTGGAGTCCA
CGTTCTTTAATAGTGGACTCTTGTTCCAAACTGGAACAACACTCAACCCTATCTCGGTCTATTCTTTTGATTTAT
AAGGGATTTTGCCGATTTCGGCCTATTGGTTAAAAAATGAGCTGATTTAACAAAAATTTAACGCGAATTTTAACA
AAATATTAACGCTTACAATTTAG
